# Selective macrocyclic peptide modulators of Lys63-linked ubiquitin chains disrupt DNA damage repair

**DOI:** 10.1038/s41467-022-33808-6

**Published:** 2022-10-18

**Authors:** Ganga B. Vamisetti, Abhishek Saha, Yichao J. Huang, Rajeshwer Vanjari, Guy Mann, Julia Gutbrod, Nabieh Ayoub, Hiroaki Suga, Ashraf Brik

**Affiliations:** 1 Schulich Faculty of Chemistry, Technion-Israel Institute of Technology, Haifa, 3200008 Israel; 2 Department of Chemistry, Graduate School of Science, The University of Tokyo, Bunkyo-ku, 7-3-1 Hongo, Bunkyo, Tokyo, 113-0033 Japan; 3 Facultly of Biology, Technion-Israel Institute of Technology, Haifa, Haifa, 3200008 Israel

**Keywords:** Peptides, Screening, Ubiquitylation, DNA damage and repair

## Abstract

Developing an effective binder for a specific ubiquitin (Ub) chain is a promising approach for modulating various biological processes with potential applications in drug discovery. Here, we combine the Random Non-standard Peptides Integrated Discovery (RaPID) method and chemical protein synthesis to screen an extended library of macrocyclic peptides against synthetic Lys63-linked Di-Ub to discover a specific binder for this Ub chain. Furthermore, next-generation binders are generated by chemical modifications. We show that our potent cyclic peptide is cell-permeable, and inhibits DNA damage repair, leading to apoptotic cell death. Concordantly, a pulldown experiment with the biotinylated analog of our lead cyclic peptide supports our findings. Collectively, we establish a powerful strategy for selective inhibition of protein-protein interactions associated with Lys63-linked Di-Ub using cyclic peptides. This study offers an advancement in modulating central Ub pathways and provides opportunities in drug discovery areas associated with Ub signaling.

## Abstract (web summary)

Finding a selective modulator of Lys63-linked ubiquitin chains has proven very challenging. Here, the authors develop potent macrocyclic peptide binders of Lys63-linked di-ubiquitin chains that interrupt DNA damage repair and lead to apoptotic cell death.

## Introduction

Ubiquitination is a complex post-translational modification (PTM) and is involved in various cellular processes^1^. In ubiquitination, the C-terminal glycine of ubiquitin (Ub) is linked mainly to the ε-amine side chain of a lysine residue of a substrate protein—a process that is achieved by the action of three enzymes known as the E1-E3^2^. PolyUb chains with different linkages (e.g., Lys63-linked Ub chains) can be formed by elongation of Ub via the addition of another Ub, to one of its seven lysine residues (e.g., Lys63) or the N-terminus^3,4^. In addition, Ub monomers attached to each other through multiple types of linkages form heterotypic chains such as mixed or branched chains^5,6^. Importantly, Ub chains with different linkage types have distinct topologies and dynamics, where each Ub chain is recognized by a specific subset of cellular proteins^7^. As a result, each chain could lead to a particular cell signaling, such as proteasomal degradation (e.g., Lys48-linked Ub chains), mitophagy, cell-cycle regulation, protein trafficking, autophagy, DNA repair (e.g., Lys63-linked Ub chain), regulation of NF-κB signaling^8^ (e.g., Met1/Lys63 branched chains) and immune response^9–14^. Like most other PTMs, ubiquitination is a reversible process in which a family of enzymes known as deubiquitinases (DUBs) trims or completely detaches the Ub chain from the ubiquitinated protein^15^.

The major components of the Ub system (e.g., DUBs, E1-E3s, and 26S proteasome) are well-known targets in drug development, in which some of these already resulted in approved cancer drugs (e.g., Bortezomib)^16^. While most approaches focus on interfering with the activity of a specific enzyme involved in the Ub system^17^, a different approach has emerged to target the Ub chain itself, as the code of signaling^18,19^. Recently, we discovered a class of cyclic peptides, which bind specifically to Lys48-linked Ub chains, leading to interference with the specific DUBs as well as proteasomal degradation of ubiquitinated proteins. These cyclic peptides, which were selected using the Random Non-standard Peptides Integrated Discovery (RaPID) approach against synthetic Lys48-linked Ub chains, exhibited a high level of apoptosis and attenuated tumor growth in vivo^20–22^.

The second most predominant class after Lys48-linked Ub chains in cells are Lys63-linked chains^23^. Finding a selective modulator for the Lys63-linked chains should allow us to interfere with other biological pathways, as they are involved in non-proteolytic cellular processes, such as DNA damage repair (DDR)^14^. A major challenge with this approach stems from the structural features of the Lys63-linked Ub chain, which has been shown to adopt an opened structure in the crystal and an ensemble of conformations in solution^24^. This is vastly different from the more defined and closed conformation of the Lys48-linked Ub chains^25^. Moreover, in Lys63-linked Ub chains, the hydrophobic patches of the proximal Ub monomer and the distal Ub do not lie on the same surface, presumably making each Ub in this chain behave as a separate unit^24^. Therefore, developing specific cyclic peptide modulators that could interact with both Ub units and interfere with the function of these chains is very challenging and remained an unexplored area.

Having succeeded with Lys48-linked chains using cyclic peptide modulators, we aimed to push the boundaries of this approach and explore targeting the Lys63-linked chains. The RaPID method has gained special attention due to its ability to generate diverse libraries, using in vitro translation against a protein of interest (POI), with a unique chemical space, each composed of up to trillion thioether-macrocyclic peptides^26^. We reasoned that combining these features with our ability to synthesize any of the Ub chains with a defined length, linkage, and high purity^27^, should allow us to selectively target any of the desired chains. We apply this strategy to discover a class of macrocyclic peptides that bind specifically and in high affinity to Lys63-linked Di-Ub chains. We characterize the discovered cyclic peptide and their chemically modified analogs, resulting in a highly potent compound that is cell-permeable and disrupts DDR leading to cell death through apoptosis.

## Results and discussion

### Identification of Lys63-linked Di-Ub binders using RaPID method

**Fig. 1:**
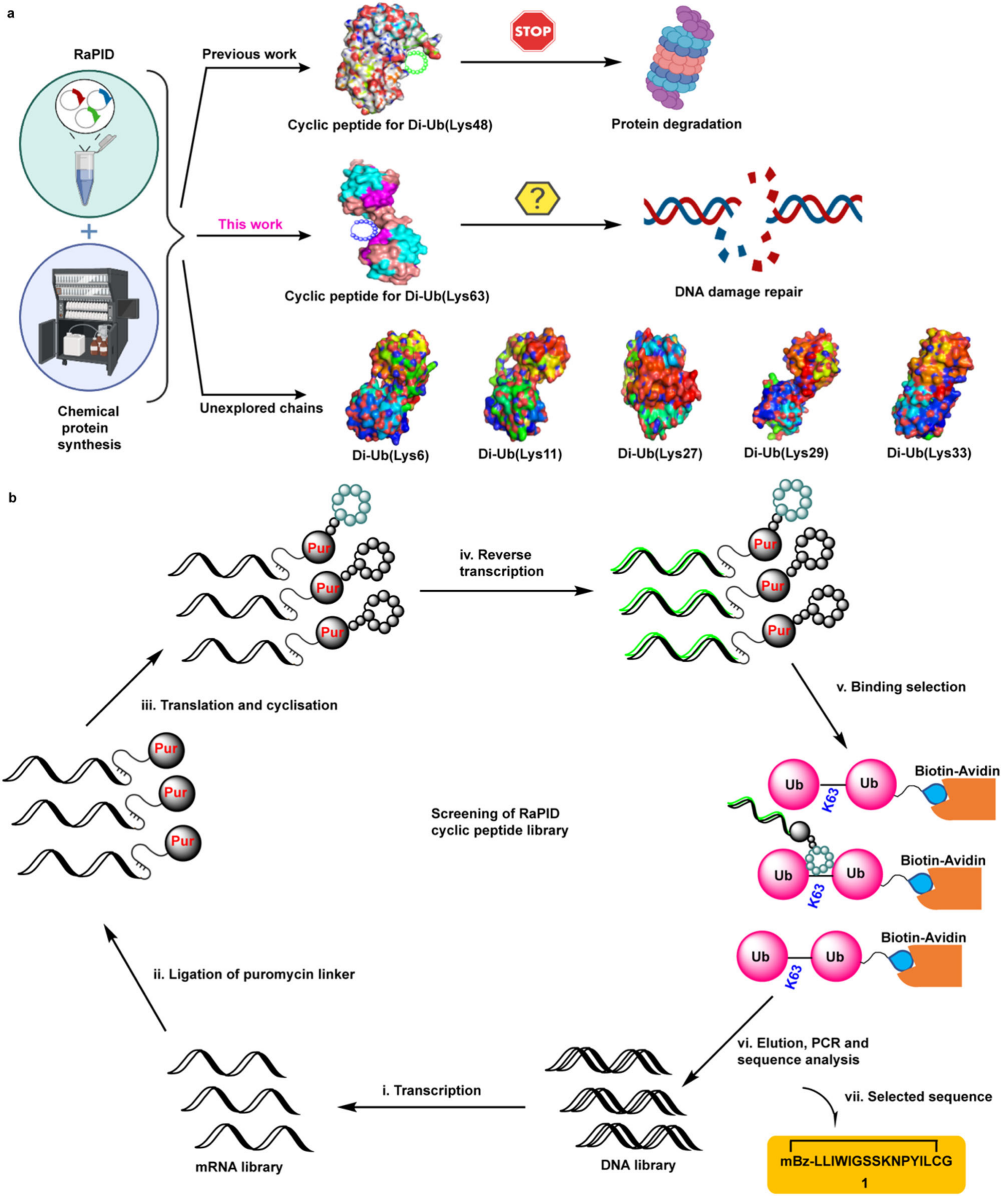
Strategy for the development of macrocyclic peptides against the Lys63-linked Ub chain. **a** The general discovery process for macrocyclic peptides for Di-Ub chains using chemical protein synthesis and RaPID approach to affect specific biological functions. **b** Schematic presentation of RaPID method for the identification of potent binder 1 (CP1) for Lys63-linked Di-Ub.

We utilized chemical protein synthesis to prepare Lys63-linked Di-Ub with a biotin tag at the N-terminus of the distal Ub (Supplementary Fig. 1)^28^. We then used this chain as a bait for the RaPID display to screen a library of trillion mRNA•cDNA-tagged cyclic peptides (Fig. 1a, b) against Lys63-linked Di-Ub, providing the first round of selection for cyclic peptides. Mono-ubiquitin (Ub1) binders were then removed by introducing an additional round of selection. After repetition of 2–5 rounds, followed by deep sequencing of the cDNA library, enabled the discovery of a highly specific and tight binding de novo cyclic peptide 1 (CP1) (Supplementary Fig. 2) with a low-nanomolar dissociation constant (K_D_) value of 16 nM, as measured by surface plasmon resonance (SPR) (Supplementary Fig. 24).

### Synthesis and screening of binders for Lys63-linked Di-Ub

**Fig. 2:**
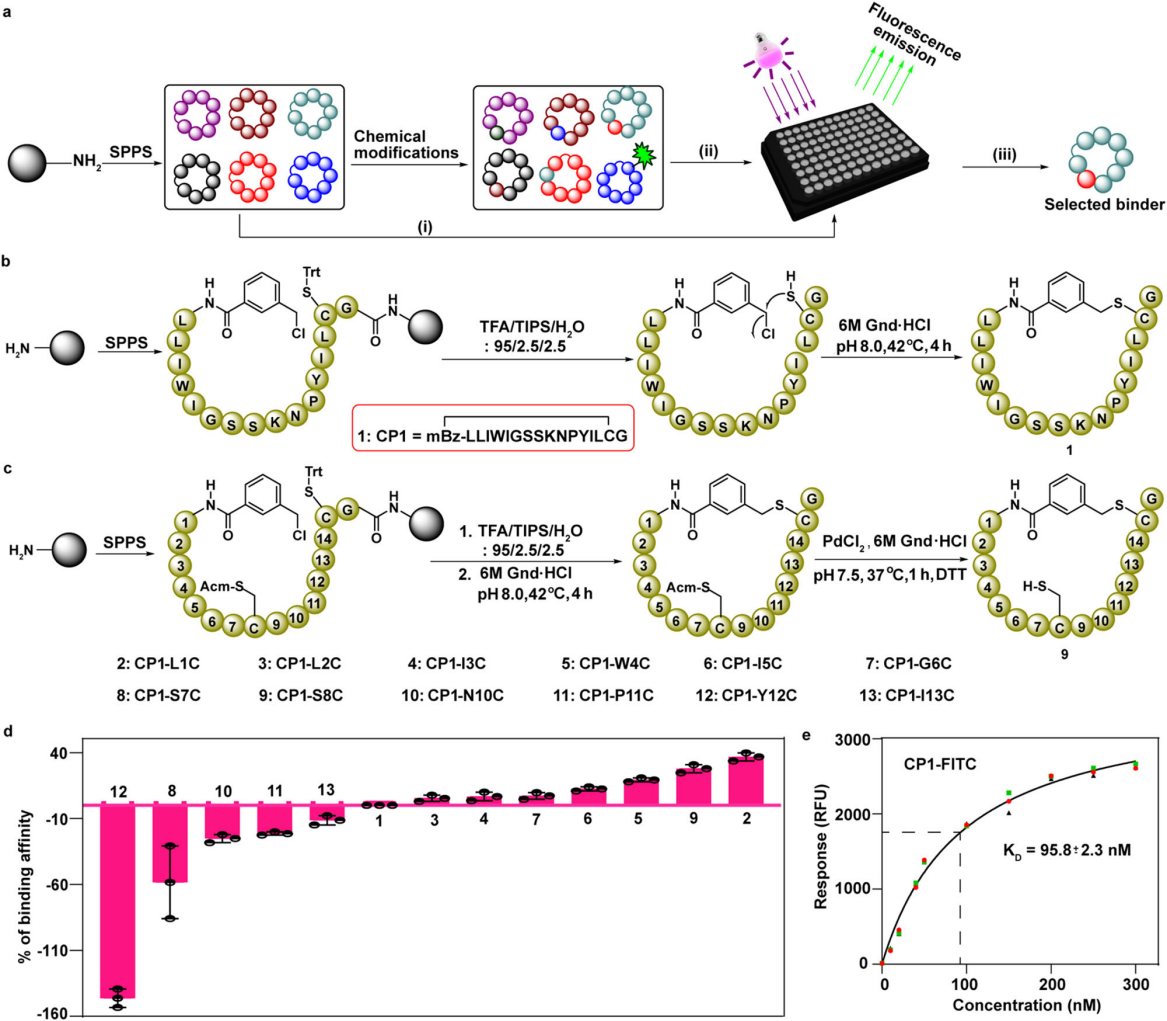
Chemical synthesis of cyclic peptide analogue of 1 and their affinity screening for Lys63-linked Di-Ub. **a** General presentation for screening of the cyclic peptide library, chemically prepared to employ Fmoc-SPPS. **b** Preparation of cyclic peptide 1 (CP1). **c** CP1 with a Cys residue at various positions in the peptide sequence. **d** The binding affinity of cyclic peptides to Lys63-linked Di-Ub, normalized to the affinity of CP1. Data were plotted as mean ± SD for *n* = 3 biologically independent experiments and each in triplicates. **e** Concentration dependent binding study for FITC-labeled cyclic peptide 1 (CP1-FITC). Mean binding response values for each concentration of CP1-FITC were taken for *n* = 3 biologically independent experiments (black, red, and green) and the binding curve (black line) determining the K_D_ (95.8 ± 2.3 nM) of CP1-FITC. All source data are provided as a Source data file.

For determining the relative binding affinity of additional chemically modified cyclic peptides based on our lead compound. we employed our recently developed fluorescence-based assay (Fig. 2a and Supplementary Fig. 3)^29^. Therefore, we prepared fluoresceine-tagged cyclic peptide 1 (CP1-FITC) (Supplementary methods) and determined a K_D_ value of 95.8 ± 2.3 nM (Fig. 2e and Supplementary Fig. 25).

In the RaPID system, peptide cyclization occurs via a selective nucleophilic attack of the thiol side chain of Cys located at the C-terminal region, on the non-amino acid initiator, such as chloroacetyl (ClAc), 3-(chloromethyl)benzoic acid (mClBz), or 4-(chloromethyl)benzoic acid (pClBz). Notably, adding any additional Cys in the sequence, when using this approach, for subsequent functionalization is not possible due to the lack of chemoselectivity in the cyclization step.

In general, the effect of the side chain in a peptide sequence is evaluated through Alanine (Ala) scanning. Here, in our current study, we performed a Cys mutation, where its free sulfhydryl group (-SH) side chain allows further modification to generate an additional library, which might help to further enhance the binding affinity for Lys63-linked Di-Ub chain and changes its physiochemical properties. To expand our library by chemical mutagenesis using chemical Cys modification, without interfering with the cyclization step, we employed Fmoc-solid-phase peptide synthesis (Fmoc-SPPS) to prepare derivatives of cyclic peptide 1 (Fig. 2b and Supplementary Fig. 4) bearing orthogonally protected Cys with acetamidomethyl (Acm) at various positions. Global deprotection and cleavage leave the trityl protecting Cys residue with a free thiol group, which undergoes cyclization with the mClBz moiety. In the following step, Acm was removed via PdCl_2_, to give a free thiol group^30^, as shown in the synthesis of a cyclic peptide 9, as a representative example (CP1-S8C), (Fig. 2c and Supplementary Fig. 5). Using the same strategy, we prepared additional derivatives of cyclic peptide 1, each having a second Cys residue at different positions (Supplementary methods).

With cyclic peptides 2–13 in hand, we then examined their affinity for the Lys63-linked Di-Ub using our fluorescence-based assay^29^. The relative binding of each cyclic peptide was normalized to cyclic peptide 1, as a reference, where the binding affinity of each cyclic peptide is inversely proportional to the FITC fluorescence of CP1-FITC. Using this assay, we identified that cyclic peptide 2 (CP2) is a more effective binder of Lys63-linked Di-Ub compared to 1 (Fig. 2d).

To further attempt improve the binding affinity of cyclic peptide 2, we utilized the advantage of the free thiol in cyclic peptide 2 for chemical modifications with different groups. Notably, the alkylation was done in a one-pot manner employing removal of Acm protecting group and treatment with various alkyl halides. This in situ reaction allowed the rapid preparation of five different alkylated (15–19) and arylated^31^ (20–21) derivatives of 2 (Supplementary Figs. 6–13). Unfortunately, all the modified peptides, showed diminished binding compared to the cyclic peptides 1 and 2 (Supplementary Fig. 14).

### Selectivity of cyclic peptide 2 for Lys63-linked Ub chains

**Fig. 3:**
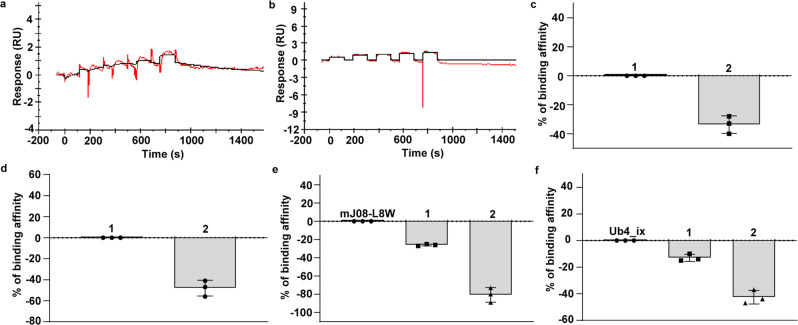
The binding affinity of cyclic peptides 1 and 2 against Ub chains with different linkages and lengths. **a** Binding of cyclic peptide 1 on Lys11 linked Di-Ub, which indicated no binding by SPR. (Red: original trace, Black: fitting curve). **b** Binding of cyclic peptide 1 on Lys29-linked Di-Ub, which indicated no binding by SPR. (Red: original trace, Black: fitting curve). **c** Relative binding of cyclic peptides 1 and 2 on linear Di-Ub. Data were plotted as mean ± SD for *n* = 3 biologically independent experiments. **d** Relative binding of cyclic peptides 1 and 2 on Lys6-linked Di-Ub. Data were plotted as mean ± SD for *n* = 3 biologically independent experiments. **e** Relative binding of cyclic peptides 1 and 2 on Lys48-linked Di-Ub. Data were plotted as mean ± SD for *n* = 3 biologically independent experiments. **f** Relative binding of cyclic peptides 1 and 2 on Lys48-linked Tetra-Ub. Data were plotted as mean ± SD for *n* = 3 biologically independent experiments. All source data are provided as a Source data file.

Since our lead cyclic peptide 1, had undetectable binding for Lys11 and Lys29-linked Di-Ub chains (Fig. 3 and Supplementary Fig. 21a, b), we further investigated the binding affinity of cyclic peptide 2 with the other Ub chains. For this, we compared the binding affinity of 2 and 1 to the reported Lys48-linked Di-Ub and Tetra-Ub binders; mJ08-L8W^22^, and Ub4_ix^21^, respectively, (Fig. 3 and Supplementary Fig. 21e, f). Our results revealed that 2 has a significantly lower binding affinity for linear Di-Ub (Supplementary Fig. 21c), Lys6-linked Di-Ub (Supplementary Fig. 21d), Lys48-linked Di-Ub, and Lys48-linked Tetra-Ub. In all these experiments, cyclic peptide 2 exhibited higher selectivity for the Lys63-linked Di-Ub chain compared to cyclic peptide 1 (Fig. 3 and Supplementary methods).

### Cell permeability study of cyclic peptide binders

**Fig. 4:**
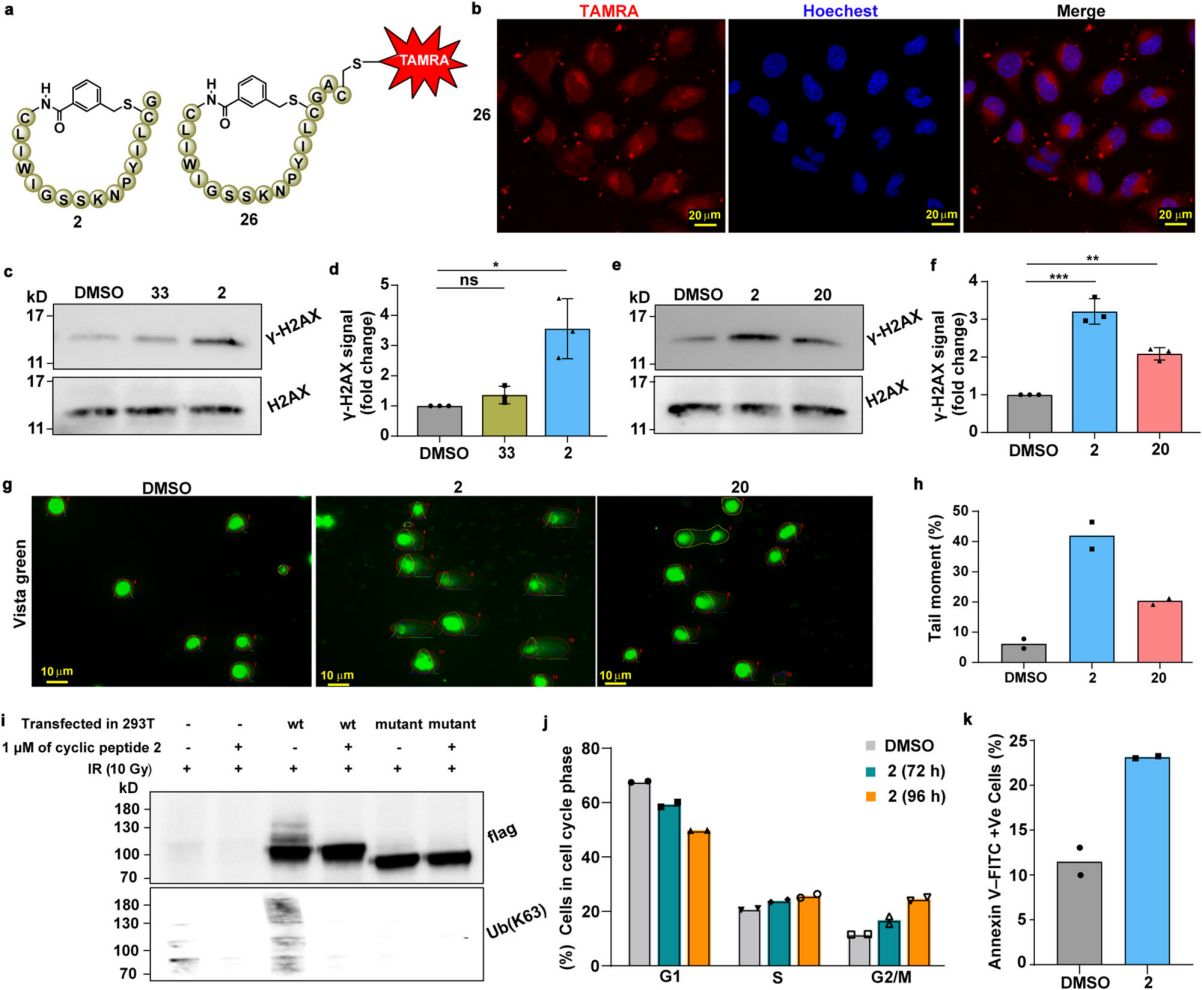
Disruption of DNA damage repair activity by cyclic peptides. **a** The chemical composition of cyclic peptide 2 and its TAMRA labeled 26. **b** Representative confocal images of 26 in live U2OS cells. Images representative of *n* = 2 biologically independent experiments. Scale bars 20 μm. **c** Western blot analysis of lysates from U2OS cells treated with 33 (a scrambled sequence of cyclic peptide 2) and 2 (upper panels). H2AX was used as a loading control (lower panels) which was performed in a different blot. Representative of *n* = 3 independent experiments. **d** Quantified relative γ-H2AX signals (**c**) for *n* = 3 independent experiments. Data are presented as mean values ± SD. (Two-tailed, unpaired *t*-test *p = 0.0112 and ns is non-significant). (DMSO: gray, 33: yellowish-green, and 2: skyblue). **e** Western blot analysis of lysates from U2OS cells treated with 2 and 20 (upper panels). H2AX was used as a loading control (lower panels) which was performed in a different blot. Representative image of *n* = 3 independent experiments. **f** Quantified relative γ-H2AX signal from (**e**) for *n* = 3 independent experiments. Data are presented as mean values ± SD. (Two-tailed, unpaired *t*-test ***p = 0.0003 and **p = 0.003). (DMSO: gray, 2: skyblue, and 20: salmon). **g** DNA damage was visualized by a “comet-like” vista green signal from the DNA of individual cells, analyzed over 100 cells in *n* = 2 independent experiments. Scale bars 10 μm. **h** Quantified relative tail moment of images (**g**) for *n* = 2 independent experiments, using “OpenComet” plugged-in to FiJi (ImageJ, open version). Data are presented as mean values (DMSO: gray, 2: skyblue, and 20: salmon). **i** Western blot using anti-flag antibody (upper panels) and anti-ubiquitin(Lys63-specific) (lower panels) from anti-flag immunoprecipitated 293T cell lysates treated with and without 2 and transfected with RFN168 wt or mutant. Representative of *n* = 2 independent experiments. **j** The cell cycle distribution of HeLa cells treated with and without 2 after 72 and 96 h. From *n* = 2 independent flow cytometry experiments (>15,000 cells each condition). Data are presented as mean values (DMSO: silver, 2 (72 h): bluish green, and 2 (96 h): orange). **k** The relative population of annexin V-FITC (apoptotic) cells after 96 h. From *n* = 2 independent flow cytometry experiments (>20,000 cells for each condition). Data are presented as mean values (DMSO: gray and 2: skyblue). All source data are provided as a Source data file.

To check the cellular effect of our cyclic peptides, we initially aimed to examine the cell-permeability of cyclic peptide 2. For this, we prepared FITC- or TAMRA-labeled peptides 2-FITC (29) and 2-TAMRA (26) (Supplementary Figs. 15–20 and 22–23) by incorporating Cys(S^t^Bu) to facilitate labeling with the maleimide dyes followed by orthogonal palladium promoted Acm removal to expose Cys1^31^. To examine the fluorophore influence on the binding of cyclic peptide 2, we determined the K_D_ value of cyclic peptides 2-FITC (29) and 2-TAMRA (26) using our fluorescence-based assay as; 43.2 ± 4.0 and 47.4 ± 5.9 nM, respectively, (Supplementary Figs. 26–28). Our results showed that the fluorophore selection (i.e., FITC or TAMRA) does not affect the binding affinity of the cyclic peptides to Lys63-linked Di-Ub. We then measured the delivery of these cyclic peptides to U2OS cells (Methods section) by laser scanning confocal microscopy (LSCM) and confirmed that 2-TAMRA (26) has an efficient cell-permeability (Fig. 4a, b and Supplementary Fig. 33). Notably, we did not observe an efficient delivery for 2-FITC (29) (Supplementary Fig. 33), suggesting that the choice of fluorescent dye significantly changes the cell-permeability behavior of the labeled cyclic peptides^32^.

### Cyclic peptide binders of Lys63-linked Ub chains disrupt DDR

After demonstrating the cell permeability of 2-TAMRA, we wondered if the unlabeled cyclic peptide 2 could modulate cellular pathways where Lys63-linked chains are known to be involved. It has been reported that Lys63‐linked Ub chains regulate various cellular events, including DNA damage repair (DDR), signal transduction, protein trafficking, and immune response. Misregulation of these pathways leads to stress, accumulation of mutations, cell cycle arrest, and apoptosis that can result in various pathological conditions^33^.

Phosphorylation of the Ser-139 residue of the histone variant H2AX, forming γ-H2AX, is the earliest known marker of double-strand breaks (DSBs)^34^. The formation of γ-H2AX is followed by the recruitment of DNA repair proteins to DSB sites. Among these proteins, RNF8 and RNF168 ubiquitin E3 ligases generate Lys63-linked Ub conjugates on histone and nonhistone proteins (e.g. 53BP1 and BRCA1) surrounding DSB sites to facilitate repair^35^. The accessibility of Lys63-linked Ub chains in these processes must be influenced by the presence of external modulators. Therefore, we expected that the interaction between our cyclic peptide 2 and Lys63-linked Di-Ub chains could disrupt the interaction between these chains and their endogenous partners and thus inhibit DSB repair, resulting in the accumulation of fragmented DNAs.

To investigate the effect of our Lys63-linked Di-Ub binder on DSB repair, we treated U2OS and HeLa cells with cyclic peptide 2 and compared the γ-H2AX levels to control cells using western blot^36,37^ (Methods section). Our results clearly show that the levels of γ-H2AX were increased by 3 and 3.2-fold upon treatment with cyclic peptide 2 in U2OS and HeLa cells, respectively. Importantly, 33 (a scrambled sequence of cyclic peptide 2, Supplementary Fig. 31), which does not have traceable binding affinity to the Lys63-linked Di-Ub, does not alter the levels of γ-H2AX (Fig. 4c, d). In addition, when we included cyclic peptide 20 (for K_D_ of FITC labeled 20 see Supplementary Fig. 29) that has a lower binding affinity to the chain compared to 2, exhibited an increase of γ-H2AX level by 1.6 and 1.8-fold in U2OS and HeLa cells, respectively (Fig. 4e, f and Supplementary Fig. 34). The increased in the γ-H2AX level indicates that the inhibition of the repair process depends on the affinity of the cyclic peptide to the Lys63-linked chains.

To further substantiate our findings, we sought to directly measure the integrity of DSB repair at a single cell level using comet assay^38^ (Methods section). Our results show that cells that were treated with cyclic peptide 2 exhibited an increase in the amount of fragmented DNA, as evident in the “comet-like” vista green signal from the DNA of individual cells (Fig. 4g). Moreover, the relative tail moment, which directly measures the degree of DNA damage^39^, shows a significant enhancement (~8-fold) in cells treated with cyclic peptide 2 compared to the control. Similarly, cyclic peptide 20 also induced DNA damage, but as in our previous experiments to a lower extent compared to cyclic peptide 2 (Fig. 4h).

The role of the Lys63-linked Ub chains in DDR is well-known. At DSB sites, RNF168 accumulates downstream of RNF8 and interacts with ubiquitylated H2A leading to the formation of Lys63-linked Ub-conjugates^40^. Also, the MIU motifs (MIU1 and MIU2) in RNF168 mediate its binding to Lys63-linked Ub chains on histones, which is necessary to promote DNA damage response^40^. To examine the direct binding activity of cyclic peptide 2 to the Lys63-linked Ub chains in cells, we overexpressed wild type (WT) or catalytically dead mutant (MIU1/MIU2 deleted) of RNF168 in 293T cells (Fig. 4i) and then treated the cells with DMSO or cyclic peptide 2 (Methods section). Immunoprecipitation and subsequent western blot analyses using antibody for flag (Methods section), showed several additional bands for WT RNF168 transfected cells, which is attributed to RNF168 and its ubiquitinated forms (Fig. 4i). This was further verified using antibodies for Ub (Lys63-specific, Lys48-specific, and non-specific) (Fig. 4i and Supplementary Fig. 35). Our results show that treatment with cyclic peptide 2, abolished these additional bands of RNF168, and conjugated Ub(Lys63). Importantly, this effect was absent in cells that overexpressed with a dead mutant of RNF168 (Fig. 4i). Moreover, the decrease in overall Ub and Lys63-linked Ub levels for treated cell lysate with 2, does not alter Lys48-linked Ub levels (Supplementary Fig. 35). Altogether, this establishes a direct binding activity of 2 and subsequent selective modulation of Lys63-linked Ub chains activity in cells.

### Investigation of cellular activities of cyclic peptide 2

Persistent unrepaired DNA damage mostly results in cell cycle arrest and apoptosis^41,42^. Therefore, we investigated the effect of cyclic peptide 2 on the cycle progression of HeLa cells that were treated with 1 μM of cyclic peptide 2 for 72 and 96 h and subjected to flow cytometric analysis (Methods section)^43^. We observed that cyclic peptide 2 treatment led to an increase in the population of cells at G2/M phase after 72 h (~5.2% and ~13.1% after 72 and 96 h, respectively), (Fig. 4j and Supplementary Fig. 37). Subsequently, we examined the rate of apoptosis in HeLa cells treated with cyclic peptide 2, using Annexin V–FITC/PI double staining kit (Methods section)^29^. Our results show that treating HeLa cells with 1 μM of cyclic peptide 2 for 96 h led to an increase of overall Annexin V positive cells (~11.7%) (Fig. 4k and Supplementary Fig. 36). These results suggest that cyclic peptide 2 treatment leads to elevated apoptosis. Altogether, we provide evidence that cyclic peptide 2 inhibits DSB repair through binding to Lys63-linked Ub chains which leads to G2/M cell cycle arrest and apoptosis.

### Cell membrane integrity study of cyclic peptides 2 and 26

To validate the cell membrane integrity at various concentrations of cyclic peptide 2 and 2-TAMRA (26), we have used a well-known SYTOX blue assay (Methods section)^44^. We did not detect SYTOX signal upon treatment to cells up to 2 μM of cyclic peptides (Supplementary Figs. 38, 39). These results support that the cell delivery of cyclic peptides does not compromise the cell membrane integrity.

### Inhibition of DUBs activity with cyclic peptides 2

We performed a well-established Deubiquitylating (DUB) activity assay of the cyclic peptide modulators to purified Lys63-linked Di-Ub (Methods section)^21^. We chose AMSH as a DUB enzyme that is specific for Lys63-linked Ub chains. Using various concentrations of cyclic peptide 2 showed that macrocycle inhibited the Lys63-deubiquitylating activity (Supplementary Fig. 40), through binding to Lys63-linked Di-Ub and therefore competing with DUBs recognition and processing of the chain.

### Proteomic analysis with biotinylated cyclic peptide 2

**Fig. 5:**
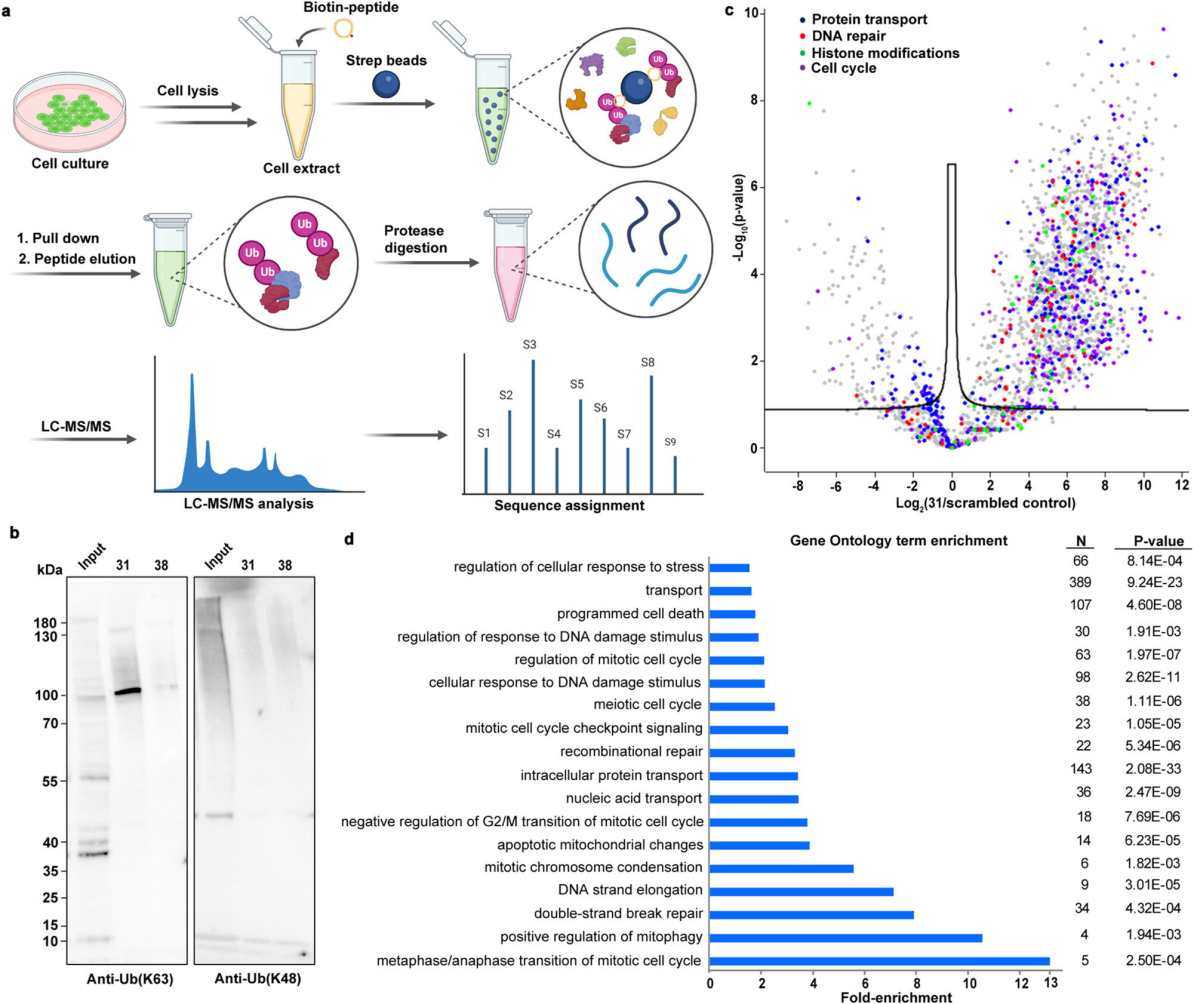
Identifying interactors of peptide 31. **a** Schematic workflow of sample preparation and analysis by proteomics using biotin-conjugated cyclic peptides. **b** Ub chains were pulled down with peptides 31 and scrambled control (38) and detected by antibodies for Lys63 and Lys48-linked Ub chains in western blot analysis. Representative of *n* = 3 biologically independent samples. **c** Volcano plot (using Perseus 1.6.7.0.) showing the differentially enriched proteins. Proteins involved in processes mediated by Lys63-linked Ub are highlighted as; protein transport: blue, DNA repair: red, histone modification: green, and cell cycle: purple. A paired t-test was performed for *n* = 3 parallel samples. **d** Gene ontology (GO) analysis of genes from (**c**) with at least threefold enrichment compared to scramble control 38 (two-tailed, unpaired *t*-test, and *p*-values were given in the plot). All source data are provided as a Source data file.

To identify ubiquitinated proteins modified via Lys63-linked Ub chains that bind to cyclic peptide binders, we modified cyclic peptide 2 with an S-biotinylation reagent, generating 31 (Supplementary Fig. 30), for the pulldown experiment (Methods section). We utilized derivative 31 and streptavidin beads to enrich its binders (which could be Lys63-linked Ub chains modified with its binder protein or protein complex, and/or other off-targets) from U2OS lysate, including the biotinylated scrambled peptide 38 (Supplementary Fig. 32) as a nonspecific control. These binders were separated by sodium dodecyl sulfate-polyacrylamide gel electrophoresis (SDS-PAGE), followed by western blot analysis with antibodies for Lys63-linked and Lys48- linked Ub chains (Fig. 5b). Cyclic peptide 31 specifically enriched proteins modified with Lys63-linked Ub chains (relative band intensity quantified using FiJi (ImageJ, open version), Supplementary Fig. 41), which supports the specificity of cyclic peptide 2 for Lys63-linked Ub chains binding. Moreover, performing an additional western blot analysis of the pulled-down components treated with DMSO or biotinylated cyclic peptides 2 (31) and comparing signal of the prominent band near 100 kDa with the input of the cell lysate and the flow-through of 31 (Supplementary Fig. 42), suggests that ~100 kDa band might represent Lys63 ubiquitinated proteins. Importantly, the pull-down samples do not contain native DNA(s) that has binding affinity to Lys63-linked Ub chains present in protein lysate, may be due to applied proteolysis conditions for eluting binders from beads (Supplementary Fig. 43).

To identify the proteins enriched by 31, we performed on-bead digestion followed by label-free proteomics (Fig. 5a and Methods section)^45^. We enriched a significant amount of proteins (~1100), in which a substantial number of proteins (~450) are involved various cellular processes where Lys63-linked Ub chains are primarily involved (e.g., DNA repair, transport, cell cycle, and histone modifications), shown by the color dots in the volcano plot (Fig. 5c). We have identified 68 improved terms of DNA repair proteins enriched by 31, shown in a cluster form with string networks (Supplementary Fig. 44 and Supplementary Table 2). Importantly, we have enriched a few proteins that have specific affinity to Lys63-Ub chains, such as UBR5, PCNA, BABAM1, and PSMD14. The gene ontology analysis showed enriched GO terms for DSB repair, regulation of mitotic cell cycle, mitotic chromosome condensation, transport, and response to stress, and others (Fig. 5d), suggesting that 31 exclusively pull-down proteins attached to Lys63-linked Ub chains. All these results together implying that the cyclic peptide 2 specifically bind to Lys63-linked Ub chains and regulates cellular processes like DDR, cell cycle, etc., in which Lys63 chain type is predominantly involved.

We discovered macrocyclic modulators of Lys63-linked Di-Ub by combining chemical protein synthesis, RaPID selection, and late-stage modifications. We exploited the power of chemical synthesis to produce an additional library of Cys mutants and their modified analogs. This multidisciplinary screening approach produced efficient cyclic peptides binders that distinguish between different Ub chain types and tightly bind to Lys63 chains. This discovery is remarkable considering the flexible and opened structure of the Lys63 chain in solution^24^. Importantly, the effective cyclic peptide does not bind to the linear Di-Ub chain, which has a similar structural feature to that of Lys63-linked Di-Ub^46^. The molecular basis for such selectivity and the mode of interactions of our cyclic peptides with the Lys63-linked chain remains to be determined, which would further enable further modifications for improving their functional properties. We observed that a slight structural modification in cyclic peptide-based Ub-binders affects extensively the binding efficiency. This highlights the strength of our approach in selecting specific Ub-chain binders, despite subtle differences at the molecular level, for potential drug development.

Our discovered cyclic peptide is a cell-permeable modulator of the DDR pathway which leads to DNA damage accumulation, cell cycle arrest in G2/M phases, and apoptosis. Moreover, proteomic analysis of proteins that were enriched with our cyclic peptide revealed crucial elements of the DDR pathway involving the Lys63-linked Ub chain.

Current therapeutic approaches to target components of the ubiquitination machinery focus on inhibiting the activity of a specific enzyme. As the vast majority of human cancers have a defective repair pathway, targeting the remaining functional repair pathways by modulating Lys63-linked Ub chains has great therapeutic potential. Modulating DNA damage repair via targeting Lys63-linked Ub chains is an unique approach that has never been tested before. Furthermore, the absence of pharmacological inhibitors of Lys63-linked Ub chains E2-E3 enzymes, highlight the urgency for establishing innovative approaches such as targeting Lys63-linked Ub chains. While many therapeutic approaches suffer from acquired drug resistance, we believe that targeting the conserved Lys63-linked Ub chains might be able to escape the canonical drug resistance mechanisms.

Our approach provides additional opportunities in basic research associated with the Ub system. We envision that this macrocyclic peptide will become a valuable tool to modulate Ub signaling and DNA damage. The selective inhibition of Lys63-linked polyubiquitin chains by cyclic peptides could be a promising strategy for cancer therapy.

## Methods

### Materials

Peptides are synthesized by solid-phase peptide synthesis (SPPS) approach using an automated peptide synthesizer (CS336X, CSBIO) or manually in teflon filter equipped syringes, purchased from Torviq. All used chemicals are analytical grade unless specified. Palladium (II) chloride (PdCl_2_), 4-bromomethyl-7-methoxycoumarin, Dimethyl sulfoxide (DMSO), 4-(2-Hydroxyethyl)piperazine-1-ethanesulfonic acid (HEPES), 2-Iodoacetamide (IAA), Decafluorobiphenyl (Reagent grade), Hexafluorobenzene (Reagent grade), Benzyl bromide (Reagent grade) were purchased from Sigma-Aldrich. Trifluoroacetic acid (TFA), Dichloromethane (DCM), Diisopropylethylamine (DIEA), and N, N-dimethylformamide (DMF) were purchased from Biolab. Tetramethylrhodamine-5-maleimide (TAMRA) and Fluorescein-5-Maleimide (FITC) were purchased from Thermo Fisher Scientific. 3-(chloromethyl)benzoic acid, 2-Chloroacetic acid were purchased from Acros Organics. Tert-butyloxycarbonyl (Boc), 9-fluorenylmethoxycarbonyl (Fmoc) protected amino acids were purchased from GL Biochem. Resins were purchased from CreoSalus. Dithiothreitol (DTT) and Triisopropylsilane (TIPS) were purchased from Alfa Aesar. All coupling reagents [(6-chlorobenzotriazol-1-yl)oxy-(dimethylamino)methylidene]-dimethylazanium hexafluorophosphate (HCTU), 1-[bis(dimethylamino)methylene]−1H-1,2,3-triazolo[4,5-b]pyridinium 3-oxid hexafluorophosphate (HATU), and Hydroxybenzotriazole (HOBt) were purchased from GL Biochem and Luxembourg Bio Technologies. A Thermo instrument (Dionex Ultimate 3000) using Xbridge (4.6 × 150 mm, 3.5 µm, BEH300 C4, waters) column was used for analytical high-performance liquid chromatography (HPLC) with 1.2 ml/min flow rate. Thermo Scientific instrument (Dionex Ultimate 3000) used Jupiter C4 (250 × 10 mm, 10 µm, 300 Å, Phenomenex) for semi-preparative hulk with 4.0 ml/min flow rate. Thermo Scientific instrument (Dionex Ultimate 3000) used Jupiter C4 (250 × 22.4 mm, 10 µm, 300 Å, Phenomenex) for preparative HPLC with 15.0 ml/min flow rate. All the peptides were purified by HPLC and characterized by mass spectrometry.

Dulbeccoʼs modified eagleʼs medium (DMEM), Fetal bovine serum (FBS), L-Glu, antibiotics, (penicillin/streptomycin), Trypsin/EDTA, and Phosphate-buffered saline (PBS) were purchased from biological industries. Trans-blot turbo (0.2 um PVDF) membrane for blotting and electrophoresis set-up were purchased from Bio-Rad. Pierce™ Iodoacetic Acid, Protease Inhibitor Cocktail (EDTA-free), FxCycle™ PI/RNase Staining Solution, SYBR Safe DNA Gel Stain, SYTOX® Blue, Hoechst 33342 solution (20 mM), Imperial blue strain, Cell culture plates, and High-capacity streptavidin agarose resin were purchased from Thermo-fisher. A non-protein Instant Block buffer for western blotting application was purchased from Gene Bio-Application L.T.D. NP-40 Alternative, MOPS SDS running buffer powder, mPAGE™ 4–12% Bis-Tris Precast Gel, Agarose, and immobilon Crescendo Western HRP substrate was purchased from Millipore. The comet assay kit (ab238544) was purchased from Abcam. MEBCYTO Apoptosis kit was purchased from medical and biological laboratories co. LTD. µ-Slide 8 well for live-cell confocal microscopy was purchased from Ibidi and poly-lysine hydrobromide, Benzonase Nuclease, N-methylmaleimide (NMM), Tris-base, Dithiothreitol, and β-glycerophosphate were purchased from Sigma-Aldrich. A/G PLUS-Agarose beads were purchased from Santa Cruz Biotechnology. The details of the following antibodies, recombinant rabbit monoclonal phospho-Histone H2A.X (phospho Ser139), recombinant rabbit monoclonal Histone H2A.X, rabbit monoclonal Ubiquitin (linkage-specific Lys63), rabbit monoclonal Ubiquitin (linkage-specific Lys48), secondary goat anti-rabbit or anti-mouse IgG (HRP), recombinant mouse monoclonal Ubiquitin (P4D1), and recombinant mouse monoclonal FLAG M2 used in this work were given in Supplementary Table 1.

### Mass spectrometry

LCQ Fleet mass spectrometer (Thermo Scientific) with an ESI source was used to perform electrospray ionization mass spectrometry (ESI-MS). Mass values of all products were mentioned with an average isotope composition. An infinite M200 fluorescence plate reader (TECAN) was used for fluorescent measurements. HEPES buffer (50 mM HEPES, 150 mM NaCl, 0.1% Tween, pH = 7.30). HPLC mobile phases: buffer A: 0.1% TFA in H_2_O and buffer B: 0.1% TFA in CH_3_CN.

### Cell culture procedure

HeLa (CCL-2™, ATCC) and HEK293T (293T-CRL-3216™, ATCC) cells were cultured in DMEM (high glucose) supplemented with 10% FBS, 0.2  mM L-Gln, and antibiotics (penicillin/streptomycin) in a humidified 37 °C incubator at 5% CO_2_. U-2 OS (HTB-96™, ATCC) cells were cultured in DMEM (low glucose) supplemented with 10% FBS, 0.2 mM L-Gln and antibiotics (penicillin/streptomycin) in a humidified 37 °C incubator at 5% CO_2_. To detach cells from culture flasks, the media was aspirated, and the flask was washed with sterile calcium and magnesium-free PBS before cells were treated with 0.25% trypsin 0.02% EDTA solution and returned to the incubation chamber for 4–5 min. Trypsin was quenched by adding the supplemented media. The cell suspension was collected and pelleted (2 min at 1000 × *g*). Media then aspired and the cell pellet was resuspended in fresh media. The cell density was determined using an automated cell counter (Countess II, Invitrogen) and seeded accordingly.

### Cell uptake studies

Cells were seeded for 24 h on Ibidi 8 well µ-slides treated with poly-L-lysine to reach ~90% confluency. Cells were then washed three times with warm PBS followed by incubation for 1 h with a warm serum-free medium (DMEM) containing peptides. Thereafter, cells were washed two times with warm PBS. Prior to imaging, cells were washed with an optical culture medium and stained with Hoechst (2 µg/ml). Live cell CLSM images were captured using Confocal Zeiss LSM 710 equipped with 40× NA 1.2 water immersion objective lens using a 1 AU pinhole settings. Different lasers were used for the different tags (Hoechst, TAMRA, FITC), and during analysis, the µ-slide was kept at 37 °C in a humidified chamber. Images were analyzed using ZEN 3.2 (blue edition).

### Induction of histone H2AX phosphorylation

Accumulation of histone H2AX phosphorylated at serine-139 (γ-H2AX) was studied by western blot using standard lysis protocol^47^. Briefly, treated U2OS or HeLa cells (3 × 10^6^) with samples (2 μM cyclic peptide or DMSO) were harvested and lysed in 100 μl of hot lysis buffer (50 mM Tris–HCl buffer, pH 8.0, 150 mM NaCl, 0.5% Nonidet P-40, 2 mM EDTA, and 1 mM phenylmethylsulphonyl fluoride) for 1 h on ice. Samples were then incubated for 15 min at 95 °C, then samples were again incubated with Benzonase Nuclease at 37 °C for 20 min. Solutions were centrifuged at 11,500 × *g* for 20 min at 4 °C. Cell lysates containing 20 μg of whole-cell protein were separated on 12 % SDS-PAGE gels and blotted onto polyvinylidene fluoride (PVDF) transfer membranes. After blocking with a non-protein InstaBlock buffer, the membrane was incubated overnight at 4 °C with anti-γ-H2A.X (phospho S139), a primary antibody against phospho-H2AX (1:1500). After washing with tris-buffer saline tween20 (TBST) 3 times, secondary anti-rabbit IgG (HRP) antibody dilution of 1:20,000 treated for 2 h at RT. Finally, crescendo western HRP solution was added to the membrane, and blots were imaged in the Fusion-400 ECL detection system. The γ-H2AX signal at 15 kDa in the blot was quantified using FiJi (ImageJ, open version). Anti-H2A.X was used as a loading control with dilution of 1:1500 treated for overnight at 4 °C (15 kDa and comes from the same source as γ-H2AX ‘rabbit’) (antibody information provided in Supplementary Table 1).

### DNA damage study by comet assay

Sample and slide preparation: The Comet Assay was performed following the protocol provided by Abcam “Comet Assay Kit (ab238544)” with few modifications. In brief, U2OS cells were cultured and treated with peptides similar to the previous conditions (above). After sample treatment in serum-free medium, the cells were removed from 60 mm dish by scraping. Thereafter to get the cell pellet, the cell suspension was transferred to a conical tube and centrifuge (2.01 × 10^−4^ g for 3 min) and was washed twice with ice-cold in ice-cold phosphate-buffered saline (PBS, without Mg^2+^ and Ca^2+^). After cell counting, cells were resuspended in ice-cold Mg^2+^ and Ca^2+^ free PBS to have 1 × 10^5^ cells/ml. For slide preparation, the low-melt comet agarose was pipetted onto the supplied 3-well comet slides to obtain a base layer and was incubated for 20 min at 4 °C. Then, the cell samples were mixed well with the comet agarose at 1:10 (v/v) at 37 °C and the suspension immediately transfer 75 µL gently onto the top of the base layer without disturbing the base layer. The slides were incubated again for 20 min at 4 °C. To avoid ultraviolet light damage to cell samples, all the procedures were performed under minimal light conditions. Alkaline Electrophoresis^48^: immobilized cells in the slide wells were lysed by alkaline lysis solution (Triton X-100 (1:100), DMSO (1:10), 2.5 M NaCl, 100 mM Na_3_EDTA, 10 mM Tris-base, pH 10). The slides with embedded cells were transferred to a container containing pre-chilled alkaline lysis solution and incubated overnight at 4 °C in dark. Then after, the solution was replaced with the pre-chilled alkaline electrophoresis buffer (300 mM NaOH, 1 mM Na_3_EDTA, pH > 13) and leftover for the next 30 min at 4 °C in the dark. The slides were directly transferred horizontally to the electrophoresis chamber and filled the chamber with pre-chilled alkaline electrophoresis solution. Then, electrophoresis was done at 1 V/cm for 30 min with a constant current setting of 300 mA. After completion of electrophoresis, the slides were horizontally transferred to a container containing pre-chilled DI H_2_O for 2 min. The slides were washed similarly twice more. Finally, the slides were horizontally transferred to a container containing cold 70% ethanol and incubated for 5 min. Consequently, removed horizontally from the 70% ethanol and allow to air dry for the next 2 h at RT. The DNA was stained with provided vista green DNA dye for 15 min in the dark at RT. The slides were then prepared for microscopy analysis. Comet analysis and quantification: The images of the cells with comets were taken by a fluorescence microscope (Axio Observer Z1 LSM 700, Zeiss) with a 63× Plan-APOCHROMAT 63×/1.4 oil DTC objective (Zeiss) and a camera (AxioCam MRm, Zeiss). The ‘Tail Moment’ has been suggested to be an appropriate index of induced DNA damage in considering the migration of the genetic material. The tail moment intensity profile was analyzed using the “OpenComet” software plugged-in to FiJi (ImageJ, open version). The fluorescence signals of at least 100 cells per data point were considered for the estimation.

### Transfection of flag-tagged- wt or mutated RNF168 genes to cells and immunoprecipitation (IP) against flag antibody

*pcDNA3-Flag-RNF168* (wild-type) and *pcDNA3-Flag-RNF168 delta MIU1/MIU2* (mutant) (purchased from addgene.org, ID: 133976 and 133981) were expressed and amplified by reported literature protocol^40^. Both genes were overexpressed in Human Embryonic Kidney 293T cells. The transfection was proceeded using polyethylenimine (PEI) reagent for twelve hours. Following transfection, cells were treated with 1 μM of cyclic peptide 2 or DMSO for 36 h then exposed to ionizing radiation (IR) of 10 Gy using an X-ray machine (CellRad). After recovery for 6 h, cells were harvested and lysed using IP buffer (50 mM HEPES, pH 7.4, 100 mM NaCl, 0.5% NP-40, 10 mM EDTA, 20 mM beta-glycerophosphate) containing buffer supplemented with protease inhibitors. Finally, proteins were immunoprecipitated using flag antibody. In this, protein A/G PLUS-Agarose beads (purchased from Santa Cruz) were washed and blocked at 4 °C for 2 h in IP buffer containing 5% BSA, and whole-cell extracts were prepared using NP-40 lysis buffer and precleared. Then, performed western blot analysis (used 4–12% Bis-Tris gel and MOPS buffer used as running buffer), with primary antibody for flag; dilution of 1:1000, Ub(Lys63-specific); dilution of 1:2000, Ub(Lys48-specific); dilution of 1:1500, and Ub(P4D1); dilution of 1:1000 treated for overnight at 4 °C. After washing the blot with tris-buffered saline tween 20 (TBST), secondary anti-rabbit or anti-mouse IgG (HRP) dilution of 1:20,000, was treated for 2 h at RT. Finally, crescendo western HRP solution was added to the membrane, and blots were imaged in the Fusion-400 ECL detection system (antibody information provided in Supplementary Table 1).

### Apoptosis study using Annexin V–FITC/PI double staining method

Sample preparation: Apoptotic cell death was estimated by using the standard MEBCYTO® Apoptosis Kit (MBL) protocol^29^. In brief, HeLa cells, seeded were treated with samples (1 μM of peptide 2 or DMSO) for 96 h at 37 °C with 5% CO_2_. After sample treatment, the cells were harvested from the 60 mm dish by trypsinization and centrifuge at 2.01 × 10^−4 ^g for 4 min. The cells were washed once with phosphate-buffered saline (PBS, without Mg^2+^ and Ca^2+^) and were resuspended in supplied binding buffer subsequently stained with Annexin V-FITC and propidium iodide (PI). The annexin V–FITC positive cells were considered as apoptotic cells moreover, the early and late apoptotic cells were distinguished by negative and positive PI signals, respectively. We could not proceed with a higher concentration of the cyclic peptides due to the solubility issues in the buffer medium. Flow cytometry measurement: The different populations were analyzed using a CYTEK Aurora flow cytometer. The final fluorescence of Annexin V-FITC was obtained in 20,000 cells for the sample analysis in each independent repetition. The unstrained and single strained cells were analyzed as a reference control. All the treated samples were unmixed after measurement. Finally, the unmixed data files were analyzed using FCS Express software (version 6). Gating: For acquisition, obtain spectral information by moving the polygon gate on the FSC vs SSC plot to include the population of interest and removes cellular debris during the analysis and FSC-A versus FSC-H plot removes the cell doublets during the analysis. SPECTROFLO®SOFTWARE (version 3.0.3) sets the default gate near or on the peak emission channel. All are consistent throughout the experiment.

### Cell cycle analysis

Cell cycle analysis was performed using FxCycle™ PI/RNase Staining Solution^49^. HeLa cells were treated similarly as previously described for the apoptosis study. Following detachment, the cells were washed twice with cold PBS and then fixed with 70% ice-cold ethanol at −20 °C overnight. The fixed cell pellets were washed twice with cold PBS and then incubated with PI/RNase staining solution on ice for 30 min in the dark. Finally, flow cytometry (CYTEK Aurora) analysis of cell cycle was carried out to check the event of cell arrest considering 15,000 cell count for all the samples. The cells at various cell cycle phases were quantified using standard heat plot analysis using FCS Express software (version 6). Gating: For acquisition, obtain spectral information by moving the polygon gate on the FSC vs SSC plot to include the population of interest and removes cellular debris during the analysis and FSC-A versus FSC-H plot removes the cell doublets during the analysis. SPECTROFLO®SOFTWARE (version 3.0.3) sets the default gate near or on the peak emission channel. All are consistent throughout the experiment.

### Cell membrane integrity analysis using SYTOX™ blue assay

To assess the cell toxicity due to the loss of membrane integrity within the concentration range used for efficient live-cell delivery of synthetic cyclic peptide molecules, we proceed similarly to the cell uptake experiment and stain the cells with SYTOX™ Blue (100 nM)^44^. SYTOX™ blue dead cell stain is a high-affinity nucleic acid stain that easily penetrates cells with compromised plasma membranes but will not cross uncompromised cell membranes. This experiment showed that the used concentration of cyclic peptide 2 and 2-TAMRA (26) up to 2 μM in the cellular experiments, was in the viable range and it did not compromise the cell membrane integrity during cell penetration (Supplementary Figs. 38, 39). CLSM images were analyzed using ZEN 3.2 (blue edition).

### Deubiquitylating (DUB) inhibition activity analysis

In vitro deubiquitylating (DUB) inhibition activity analysis was performed using TRIS buffer solution (25 mM Tris-base, 150 mM NaCl, 10 mM DTT, pH 7.4)^21^. To this buffer, 2 μM of Lys63-linked Di-Ub, and cyclic peptide 2 at various concentrations (0.5-10 μM) were added and incubated at 37 °C for 30 min. To this reaction mixture, 1 μM deubiquitinase enzyme, AMSH was added which was preactivated with 20 mM DTT for 15 min at rt. Finally, the reaction mixtures were incubated at 37 °C for 30 min. The substrate without enzyme was also incubated similarly. After completing the reaction time, all the reactions were quenched by mixing them with 3X sample buffer. Then, performed 4–12% Bis-Tris gel was run using MOPS as running buffer at 200 V, and then the Gel was transferred and immunoblotted with anti-ubiquitin polyclonal mouse anti-Ub(P4D1) in a 1:1000 dilution. Goat pAb to Ms HRP IgG (HRP) conjugate was used as the secondary antibody in a 1:20,000 dilution. Finally, crescendo western HRP solution was added to the membrane, and blots were imaged in the Fusion-400 ECL detection system (antibody information provided in Supplementary Table 1).

### Pull down and proteomics analysis

The cell lysate suspension preincubated with biotinylated cyclic peptides 31 and 38 were subjected to pull down from streptavidin beads by utilizing standard protocol with few modifications^50,51^. In brief, U2OS cells (3 × 10^6^) were collected as cell pellets after trypsinization. Instantly, the lysis buffer (0.5% NP-40, 150 mM NaCl, 50 mM HEPES pH 7.5, 1 μM NMM, and 1 μM IAA) was added to the pellet for 30 min on ice then centrifuged 9000 × *g* for 15 min at 4 °C. The cell lysate suspension was divided equally into two parts and incubated overnight at 4 °C on a rotating wheel with the biotinylated peptide 31 or 38. The streptavidin agarose beads (High-Capacity Streptavidin Agarose Resin, Thermo Scientific) were equilibrated with lysis buffer 3 times under shaking conditions. The washed beads were added to each treated suspension and then incubated for 1 h at 4 °C. The beads were washed five times with wash buffer containing PBS pH 7.5 and used for proteolysis and mass spectrometry.

Western blot analysis of pull-down samples: The beads were washed similarly with PBS and the protein complexes were eluted by heating for 5 min at 95 °C with reducing buffer containing DTT (BioPrep). The eluted mixtures were examined by western blot using Anti-Ub (Lys63-specific) or Anti-Ub (Lys48-specific) (antibody information provided in Supplementary Table 1). For positive control, 0.5% v/v of the input was included.

Identification of the presence of DNA in the pull-down samples: To test the presence of DNA in the pull-down samples, we performed agarose gel electrophoresis of the pull-down components treated with and without Benzonase Nuclease (a nucleic acid removing enzyme). Enlightened with the previous study, DNA wasn’t detected in pull-down samples, even with the use of ultra-sensitive SYBR^TM^ Gold nucleic acid gel stain (Supplementary Fig. 43).

Proteolysis and Mass Spectrometry^52,53^: The elution of the interacting protein from the beads was done by 30 min incubation in 8 M Urea and 400 mM ammonium bicarbonate, then removed from the beads, reduced with 3 mM DTT (60 °C for 30 min), modified with 10 mM iodoacetamide in 100 mM ammonium bicarbonate (room temperature 30 min in the dark) and digested in 2 M Urea, 25 mM ammonium bicarbonate with modified trypsin (Promega), overnight at 37 °C in a 1:50 (M/M) enzyme-to-substrate ratio. The tryptic peptides were desalted using C18 tips (Top tip, Glygen) dried and re-suspended in 0.1% formic acid.

The peptides were resolved by reverse-phase chromatography on 0.075 × 180-mm fused silica capillaries (J&W) packed with Reprosil reversed-phase material (Dr Maisch GmbH, Germany). The peptides were eluted with a linear 60 min gradient of 5–28% solvent B (acetonitrile with 0.1% formic acid in water) 15 min gradient of 28 to 95% solvent B and 25 min at 95% acetonitrile with 0.1% formic acid in water at flow rates of 0.15 μl/min. Mass spectrometry was performed by Q Exactive plus mass spectrometer (Thermo) in a positive mode (*m/z* 300–1800 resolution 70,000 for MS1 and 17,500 for MS2) using repetitively full MS scan followed by high collision induces dissociation (HCD at 25 normalized collision energy) of the 10 most dominant ions (>1 charges) selected from the first MS scan. The AGC settings were 3 × 10^6^ for the full MS and 1 × 10^5^ for the MS/MS scans. The intensity threshold for triggering MS/MS analysis was 1 × 10^4^. A dynamic exclusion list was enabled with an exclusion duration of 20 s.

The mass spectrometry data of the three biological replicates were analyzed using the MaxQuant software 1.5.2.8 (S1) for peak picking and identification using the Andromeda search engine, searching against the human proteome from the Uniprot database with mass tolerance of 6 ppm for the precursor masses and 20 ppm for the fragment ions (Note: the protein sequence database was downloaded from uniprot on 7th May 2021). Oxidation on methionine ubiquitination on lysine and protein N-terminus acetylation were accepted as variable modifications and carbamidomethyl on cysteine was accepted as static modifications. Minimal peptide length was set to seven amino acids and a maximum of two miscleavages was allowed. The data were quantified by label-free analysis using the same software (S1). Peptide- and protein-level false discovery rates (FDRs) were filtered to 1% using the target-decoy strategy. Protein tables were filtered to eliminate the identifications from the reverse database, and common contaminants and single peptide identifications. Missing values were imputed by the minimal intensity of the project (18 in log2). Paired t-test analysis between the control and the experimental groups was done using Perseus software 1.6.7.0.

### Reporting summary

Further information on research design is available in the Nature Research Reporting Summary linked to this article.

## Supplementary information

Supplementary Information

Reporting Summary

## Data Availability

The mass spectrometry proteomics data have been deposited to the ProteomeXchange Consortium via the PRIDE partner repository with the dataset identifier PXD035924 [https://www.ebi.ac.uk/pride/archive/projects/PXD035924]. Source data are provided as a Source data file. All other related data are available from the corresponding authors upon request. Source data are provided with this paper.

## Source data

Source Data
