# Supplementary material for: Selective macrocyclic peptide modulators of Lys63-linked ubiquitin chains disrupt DNA damage repair
Source: Nat Commun. 2022 Oct 18;13:6174. doi: 10.1038/s41467-022-33808-6 (PMC9579194; doi:10.1038/s41467-022-33808-6)
Supplement: Supplementary file 1 — Supplementary Information [file 41467_2022_33808_MOESM1_ESM.pdf]

Supplementary Information

Selective Macrocyclic Peptide Modulators of Lys63-linked Ubiquitin Chains Disrupt DNA Damage Repair

Ganga B. Vamisetti, Abhishek Saha *et al.*

Supplementary Table 1: Antibody Information

Antibody names with the source	Product names	Dilutions	Company names	Catalog numbers	Clone numbers for monoclonals
Rabbit monoclonal phospho-Histone H2A.X (phospho Ser139)	Phospho-Histone H2A.X (Ser139) (20E3) Rabbit mAb	1:1500	Cell Signaling Technology	9718	20E3
Rabbit monoclonal Histone H2A.X	Recombinant Anti-Histone H2A.X antibody [EPR895] (ab124781)	1:1500	Abcam	ab124781	EPR895
Rabbit monoclonal Ubiquitin (linkage-specific Lys63)	Anti- Ubiquitin (linkage-specific K63) antibody (EPR8590-448)	1:2000	Abcam	ab179434	EPR8590-448
Rabbit monoclonal Ubiquitin (linkage-specific Lys48)	Anti- Ubiquitin (linkage-specific K48) antibody [EP8589]	1:1500	Abcam	ab140601	EP8589

Secondary goat anti-rabbit IgG H&L(HRP)	Goat anti-rabbit IgG H&L (HRP)	1:20000	Abcam	ab6721	-
Secondary goat anti-mouse IgG H&L(HRP)	Goat anti-mouse IgG H&L (HRP)	1:20000	Abcam	ab6728	-
Mouse monoclonal Ubiquitin (P4D1)	Ubiquitin (P4D1): sc-8017	1:1000	Santa Cruz Biotechnology	Sc-8017	-
Mouse monoclonal FLAG M2	Monoclonal ANTI-FLAG® M2 antibody produced in mouse	1:1000	Sigma	F1804	M2

Supplementary Methods

Synthesis of biotinylated-Lys63 linked Di-Ub

For the synthesis of the biotinylated-Lys63 linked Di-Ub chain, we prepared Ub building blocks 1 and 2 (biotin-Ub-MMP) using standard Fmoc-solid-phase peptide synthesis (Fmoc-SPPS) with the mentioned modifications in accordance with the reference.[1] The building blocks 1 and 2 are ligated to give 3. Then 3 is subjected to radical-mediated desulfurization following HPLC (employing C4 column and gradient flow of 0-60% B) and FPLC purification steps gave the desired native Lys63-linked Di-Ub chain 4 with high purity.

a

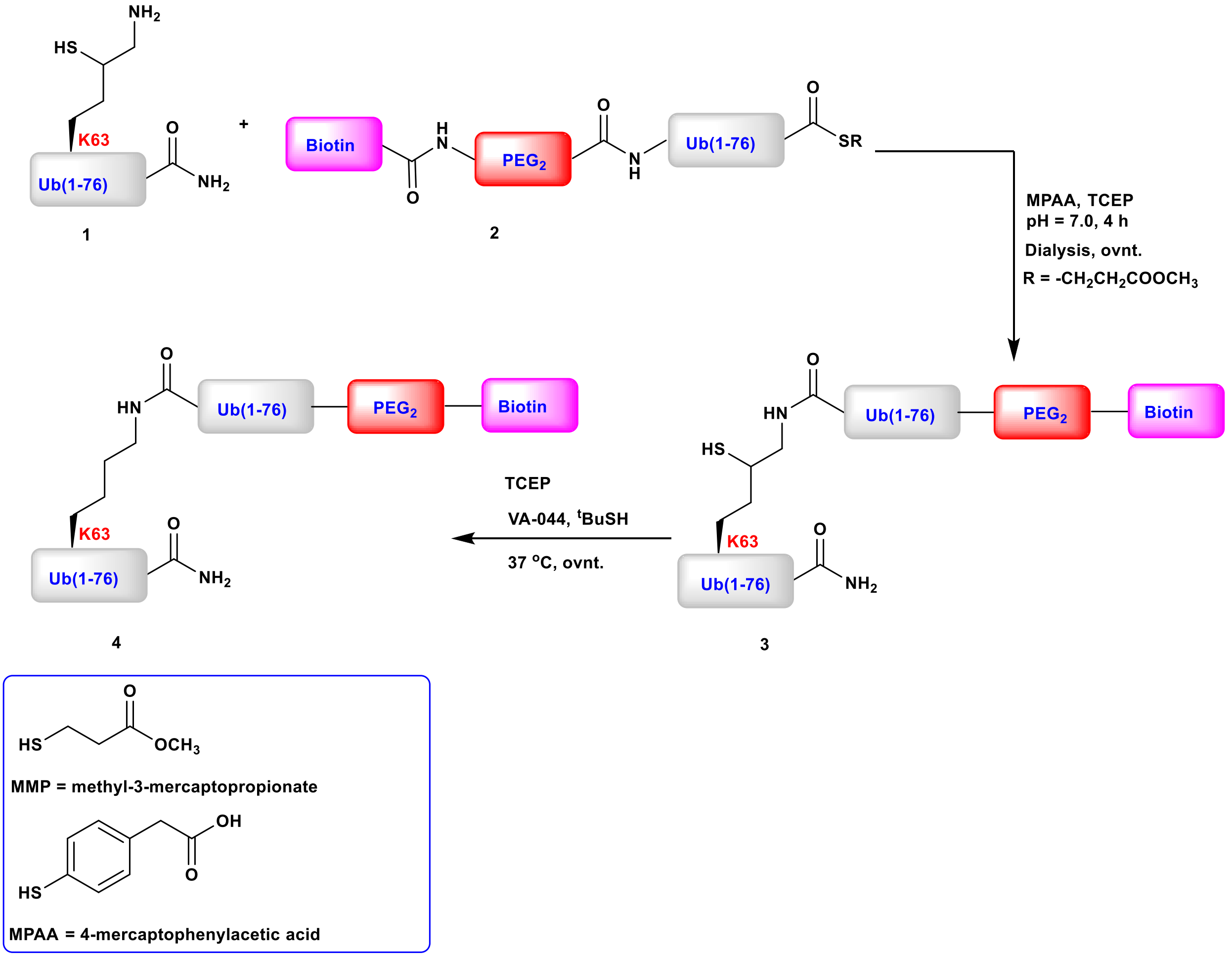

b

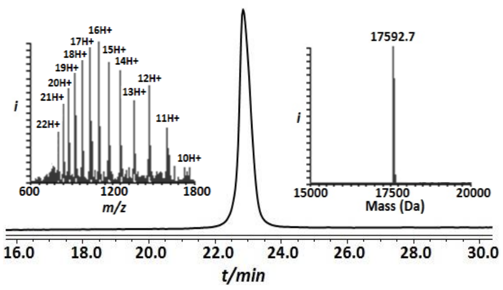

Supplementary Fig. 1 Synthesis of biotinylated-Lys63 linked Di-Ub. **a** Schematic representation for the synthesis of biotin-Lys63 linked Di-Ub. **b** Analytical HPLC and mass of the purified biotinylated-Lys63 linked Di-Ub with the observed mass 17592.7 ± 0.2 Da (calcd 17592.8, average isotopes).

Other Ub chains such as Lys6, Lys11-linked Di-Ub, Lys29-linked Di-Ub, Lys48-linked Di-Ub, Linear Di-Ub, and Lys48-linked Tetra-Ub were prepared as described previously.[2]

RaPID method for the identification of cyclic peptide 1

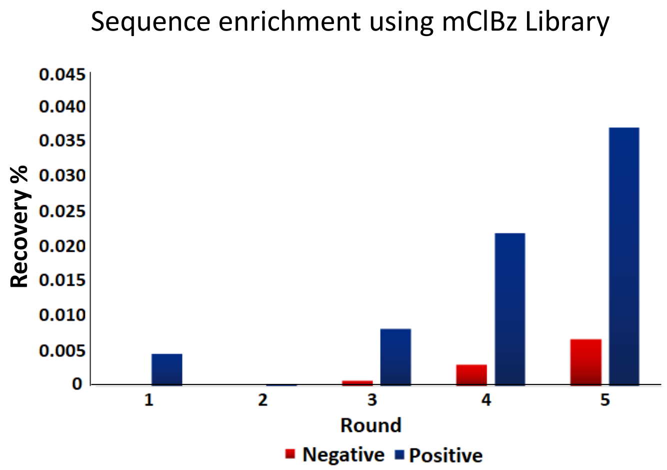

Supplementary Fig. 2 RaPID selection of three new libraries with mClBz as an initiator and its real-time PCR results. (Negative = 50% naked M280 streptavidin beads + 50% Ub1-attached M280 streptavidin beads; Positive = Lys63 linked Di-Ub-attached M280 streptavidin beads).

Experimental procedure

1. Aminoacylated tRNA synthesis: The initiator tRNA was charged with mClBz using enhanced flexible ribosome (eFX) and this is termed as $tRNA^{fMet}_{CAU.}$ Reaction conditions employed in the literature (*Chem. Eur. J.* **2020**, 26, 8022 – 8027) are applied for the preparation of tRNA solution.
2. Next, a microhelix RNA charging experiment was performed as the procedure described in the literature (*Chem. Eur. J.* **2020**, 26, 8022 – 8027).
3. Flexible *in vitro* translation (FIT): The components of the translation system employed in the literature (*Cell Chem. Biol.* **2017**, 24, 381-390) were used.
4. RaPID selection: The protocols for selection were mentioned in the literature (*Nat. Chem.* **2019**, 11, 644-652)

   Buffer selection = 1 X TBS-T (50 mM Tris, 150 mM NaCl, 0.1% Tween 20, pH 7.60).

33 µM mClBz-tRNA$^{fMet}_{CAU}$ was prepared.

5. Positive and negative readouts were collected through clone assay, where clones with P/N>20 were chosen as candidate peptides.
6. PCR amplification was performed following the extracted resulting sequence and categorized based on Python script, which gives motif corresponding to NNK library region with the correct length.

Screening of peptides using a fluorescence-based competitive assay

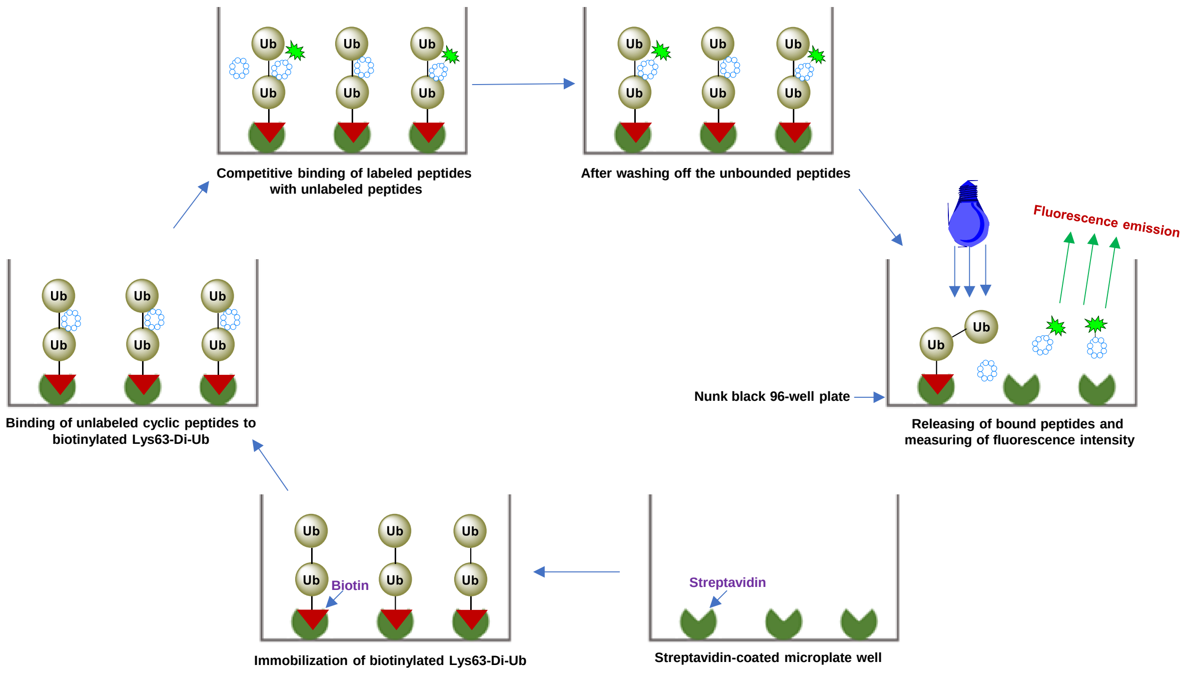

Supplementary Fig. 3 General presentation for screening the peptides using the fluoresce-based competitive assay.

As per literature procedure,[3] the streptavidin-coated microplate (96 well plate) was washed with HEPES buffer (50 mM HEPES, 150 mM NaCl, 0.1% tween, pH 7.30) and then in each well incubated 1 µg of biotinylated-Lys63-linked Di-Ub in 100 µl of the same buffer at r.t. for 30 min. Additional wells were kept without biotinylated-Lys63-Di-Ub for blank subtraction. After the washing step, peptide standard (CP1) and unlabeled peptides were incubated (5.0 molar equiv relative to biotin-Lys63-linked Di-Ub) at room temperature for 30 minutes for saturation binding to the target. Then 1 molar equivalent of FITC labeled peptide-standard (CP1-FITC) relative to biotinylated-Lys63-linked Di-Ub was incubated to compete with unlabeled peptides at r.t for 30 min. After releasing bounded peptides on treatment with 6M Gnd·HCl, the

fluorescence values are measured (λ_{ex} = 480 nm and λ_{em} = 525 nm) and these values are normalized and then calculated the relative change in binding with the below formula.[3]

$$Y = [1-(a/b)] \times 100$$

Where *Y* =Signal change relative to the standard (CP1) (in terms of %)

a = Measured signal value of each peptide candidate (in Relative Fluorescence Units)

b = Measured signal value of standard peptide (CP1).

The dissociation constant (K_D) of fluorescein-labeled peptide (CP1-FITC) was calculated in accordance with the literature [3] procedure.

Synthesis of cyclic peptide 1

Fmoc-SPPS was performed on a Rink amide resin (0.26 mmol/g, 0.1 mmol scale) for synthesizing cyclic peptide 1 as shown in Supplementary Fig. 4a. The peptide was synthesized using amino acids (4.0 equiv), HCTU (4.0 equiv), and DIEA (8.0 equiv) at room temperature. Fmoc protecting group was removed by treating the resin with 20% piperidine in DMF containing 0.1 mmol HOBt (3:5:3 min). To the N-terminal of the sequence, 3-(chloromethy)-benzoic acid was coupled as per the literature procedure.[4] The peptide was cleaved from the resin using the cocktail TFA/H_2O/TIS (95:2.5:2.5) and then precipitation in cold diethyl ether and lyophilization. The cyclization was performed by dissolving crude peptide in 6M Gnd·HCl and adjusted to pH 8.0 with NaOH followed by incubation at 42 °C for 4h. Then crude peptide was purified by using HPLC with a C4 column by using a gradient flow of 0-60% B in 60 min.

a

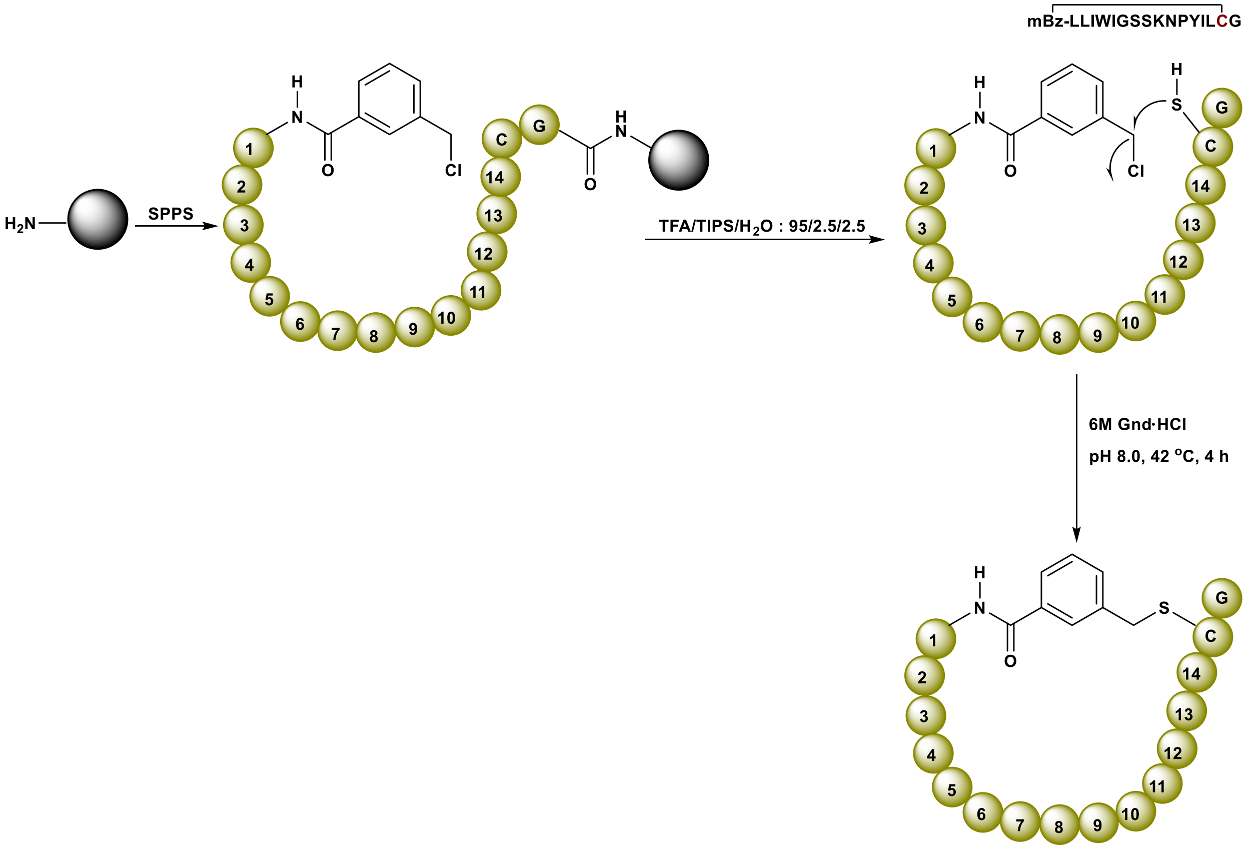

b

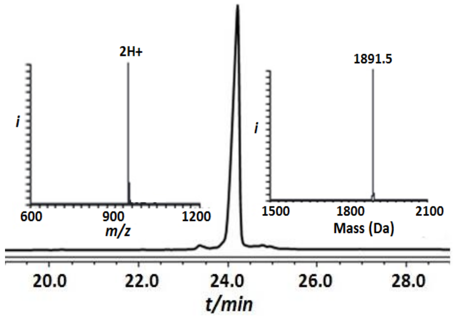

Supplementary Fig. 4 Synthesis of CP1. **a** Schematic presentation of cyclic peptide synthesis. **b** HPLC and mass analysis of cyclic peptide 1, CP1 with the observed mass 1891.5± 0.1 Da (calcd 1891.6 Da, average isotopes).

Synthesis of cyclic peptides, 2-14

Fmoc-SPPS was applied for synthesizing the cysteine mutated cyclic peptides. An orthogonally protected Cys(Acm) was incorporated at various positions of the 1 (CP1), for the synthesis of different cyclic peptides with Cys mutation. After cleavage from resin and cyclization, purification was performed (shown a representative example for the synthesis of 9). For Acm removal,[5] the peptide CP1-S8C-Acm (10.0 mg, $5.04X10^{-3}$ mmol, 1.0 equiv) was dissolved in 6M Gnd·HCl/200 mM phosphate buffer (pH 7.5, 2524.8 µl, 2 mM). Then, $PdCl_2$ (8.94 mg, 10.0 equiv) was dissolved in 100 µl of 6M Gnd·HCl/200 mM phosphate buffer (pH 7.5) at 37 °C for 10 min and it was added to the peptide solution. The reaction mixture was incubated at 37 °C for 1 h. Next, the reaction mixture was quenched with the dithiothreitol, DTT, (40.0 equiv, $2.05X10^{-1}$ mmol). After centrifugation for 5 min at 9169xg, the supernatant was injected into HPLC using a semi-preparative C4 column with a gradient flow of 0-60% B in 60 min to give free thiol-containing cyclic peptide 9, CP1-L1C (3.85 mg, 40 % yield). A similar procedure was applied for synthesizing other cyclic peptides 2-13 and cyclic peptide 14.

a

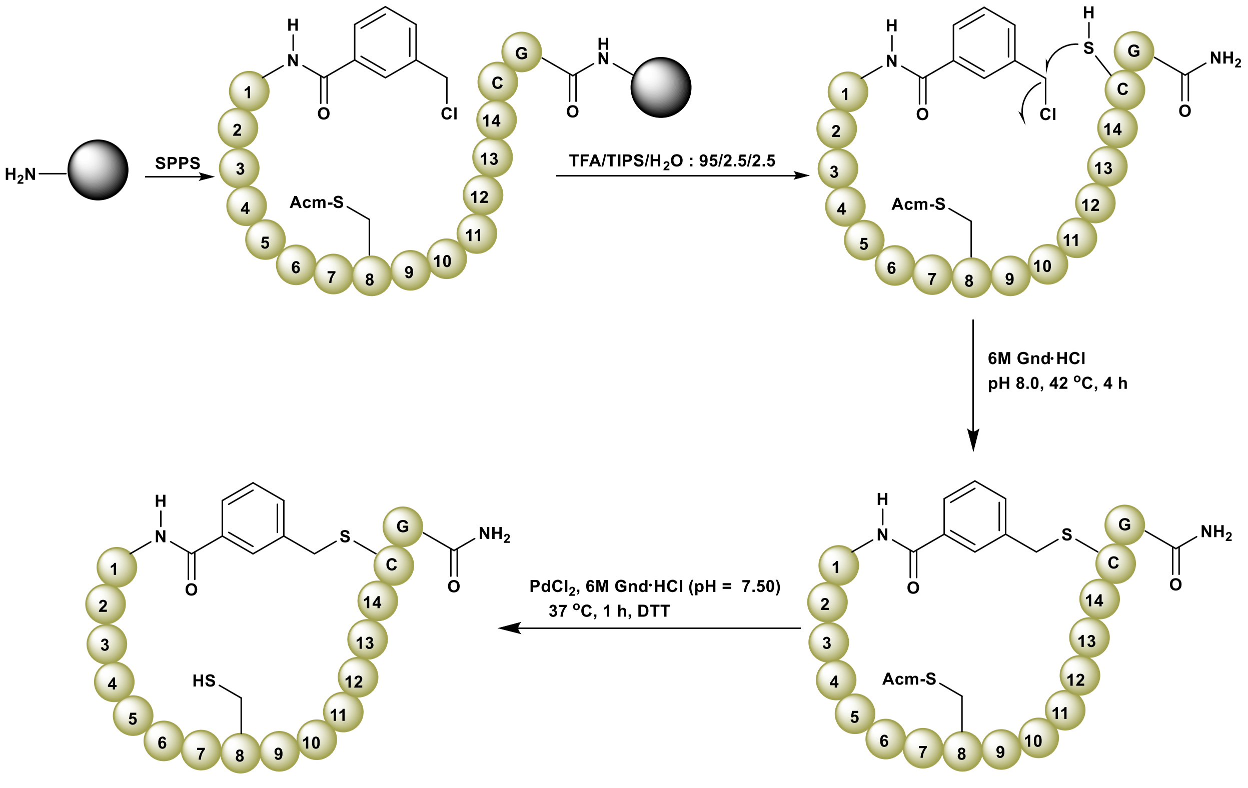

9

b

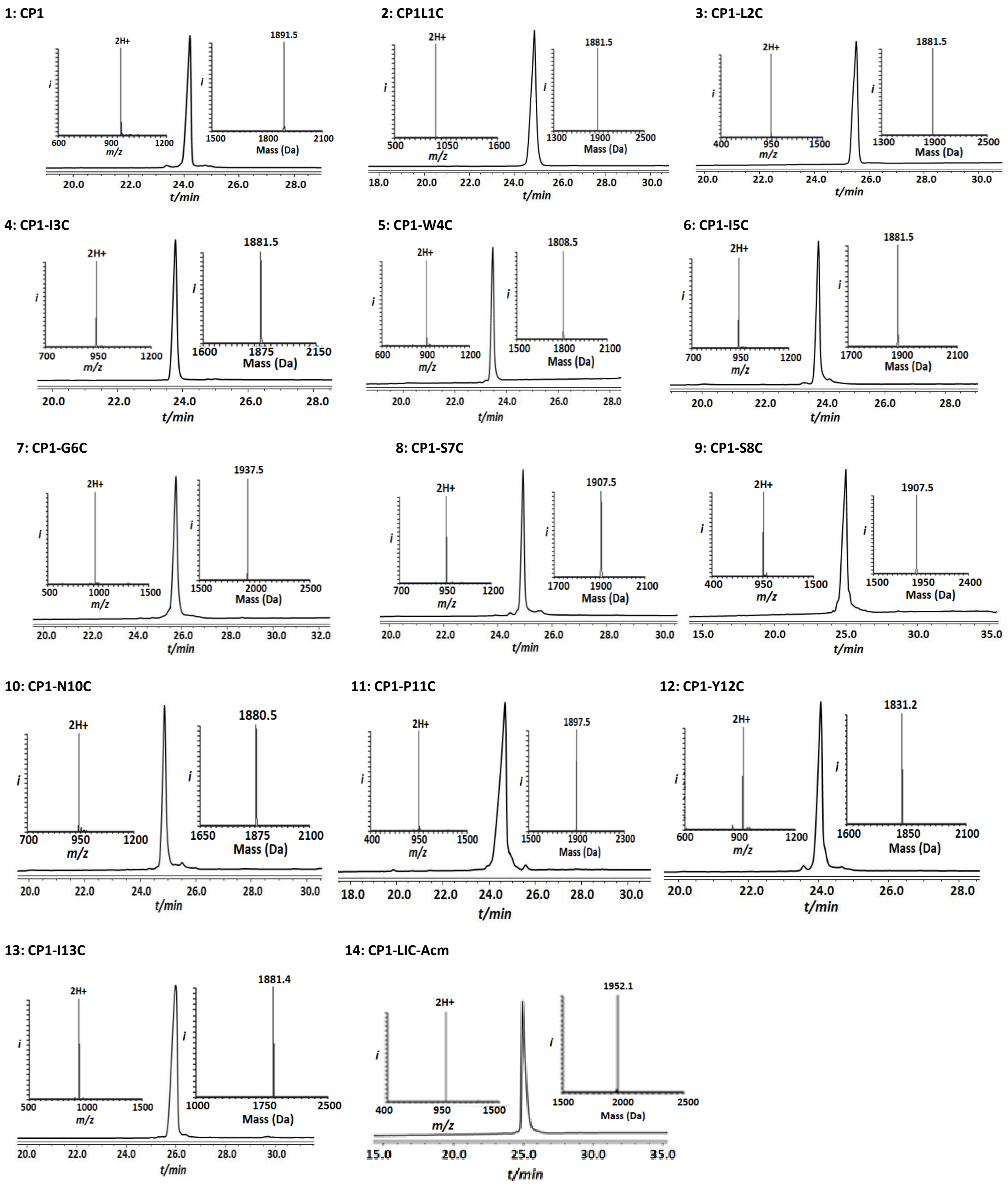
1: CP1
2H+
1891.5
Mass (Da)
m/z
t/min
2: CP1L1C
1881.5
3: CP1-L2C
1881.5
4: CP1-I3C
1881.5
5: CP1-W4C
1808.5
6: CP1-I5C
1881.5
7: CP1-G6C
1937.5
8: CP1-S7C
1907.5
9: CP1-S8C
1907.5
10: CP1-N10C
1880.5
11: CP1-P11C
1897.5
12: CP1-Y12C
1831.2
13: CP1-I13C
1881.4
14: CP1-LIC-Acm
1952.1

c Observed mass values of prepared benzyl thioether-linked cyclic peptides

S.No.	Cysteine mutated cyclic peptide	Observed mass $[M+H]^+$ (Da)	Calculated mass $[M+H]^+$ (Da)
1	CP1 = mBz-LLIWIGSSKNPYILCG , 1	1891.5 ± 0.2	1891.3
2	CP1-L1C = mBz-CLIWIGSSKNPYILCG , 2	1881.5 ± 0.2	1881.3
3	CP1-L2C = mBz-LCIWIGSSKNPYILCG , 3	1881.5 ± 0.1	1881.3
4	CP1-I3C = mBz-LLCWIGSSKNPYILCG , 4	1881.5 ± 0.2	1881.3
5	CP1-W4C = mBz-LLICIGSSKNPYILCG , 5	1808.5 ± 0.2	1808.2
6	CP1-I5C = mBz-LLIWCGSSKNPYILCG , 6	1881.5 ± 0.2	1881.3
7	CP1-G6C = mBz-LLIWICSSKNPYILCG , 7	1937.5 ± 0.1	1937.4
8	CP1-S7C = mBz-LLIWIGCSKNPYILCG , 8	1907.5 ± 0.2	1907.3
9	CP1-S8C = mBz-LLIWIGSCKNPYILCG , 9	1907.5 ± 0.1	1907.3
10	CP1-N10C = mBz-LLIWIGSSKCPYILCG , 10	1880.5 ± 0.2	1880.3
11	CP1-P11C = mBz-LLIWIGSSKNCYILCG , 11	1897.5 ± 0.1	1897.3
12	CP1-Y12C = mBz-LLIWIGSSKNPCILCG , 12	1831.2 ± 0.1	1831.2
13	CP1-I13C = mBz-LLIWIGSSKNPYCLCG , 13	1881.4 ± 0.1	1881.3
14	Acm-S CP1-L1C-Acm = mBz-CLIWIGSSKNPYILCG , 14	1952.1 ± 0.1	1952.3

Supplementary Fig. 5 Synthesis of different analogs of 1. **a** Schematic presentation of cysteine mutated cyclic peptides. **b** HPLC and mass analysis of each cyclic peptide analog. **c** Table representing the prepared benzyl thioether-linked cyclic peptides.

Synthesis of cysteine-modified derivatives of cyclic peptide 2 and their binding affinity against Lys63-linked di-Ub chain

The cyclic peptide 2, CP1-L1C, was further modified with alkylated and arylating reagents for preparing different Cys-modified cyclic peptides as described below.

(I) Chemical synthesis of cysteine modified derivatives of CP2

a

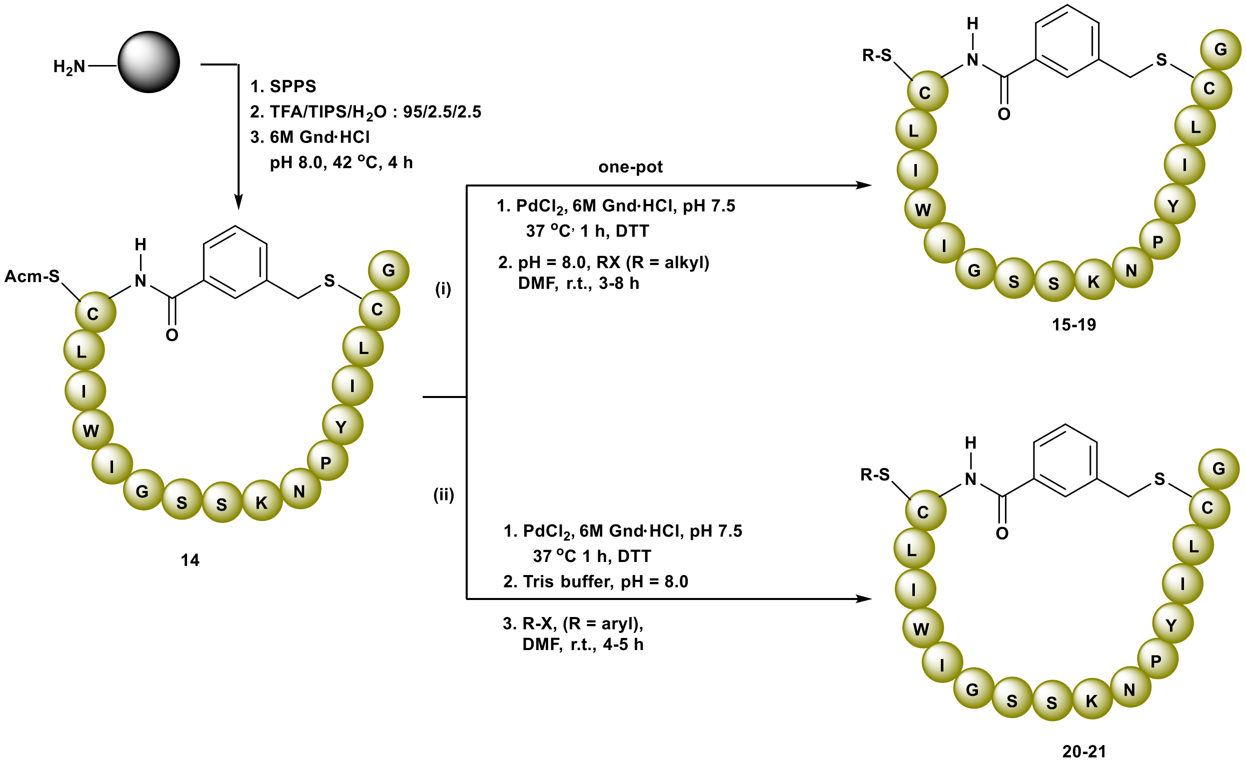

b Alkylation and arylation derivatives of CP2

Compound No.	Modified cyclic peptides
15	CP1-L1C-CH_3
16	CP1-L1C-$CH_2C_6H_5$
17	CP1-L1C-CH_2CONH_2
18	CP1-L1C-$CH_2C_{10}H_7$
19	CP1-L1C-$CH_2C_9H_5O_2$
20	CP1-L1C-C_6F_5
21	CP1-L1C-$C_{10}F_9$

Supplementary Fig. 6 Chemical synthesis of modified cyclic peptides containing Cys at position 1 (Cys1) and their affinity screening for Lys63-linked Di-Ub. **a** Schematic presentation for alkylation and arylation of Cys1. **b** The alkylation and arylation substituents of cyclic peptide 2 are presented in the table.

(I a) Synthesis of cysteine-alkylation derivatives, **15-19.**

Various cysteine-alkylated derivatives from cyclic peptide 14, CP1-L1C-Acm, were prepared in one pot as per the literature procedure.[3] The synthesis of each derivative was performed as follows.

(i) Synthesis of cyclic peptide 15, **CP1-L1C-CH_3**

Palladium chemistry is applied for Acm removal for the peptide 14, CP1-L1C-Acm (10.0 mg, $5.11X10^{-3}$ mmol, 1.0 equiv) was performed. After quenching and centrifugation (for 5 min at 9169xg) steps of the reaction mixture, separate the supernatant and adjust pH to 8.0 with NaOH. Dissolve 500 equiv of iodomethane (CH_3I) in 500 μl of DMF and was added to the reaction mixture at room temperature. The progress of the reaction mixture was monitored by HPLC using a C4 analytical column with a gradient flow of 0-60% B in 30 min. The reaction was completed within 3 h. Then the reaction mixture was purified using a preparative C4 column with a gradient flow of 0-60% in 60 min to give 15, CP1-L1C-CH_3 (4.27 mg, 44% yield).

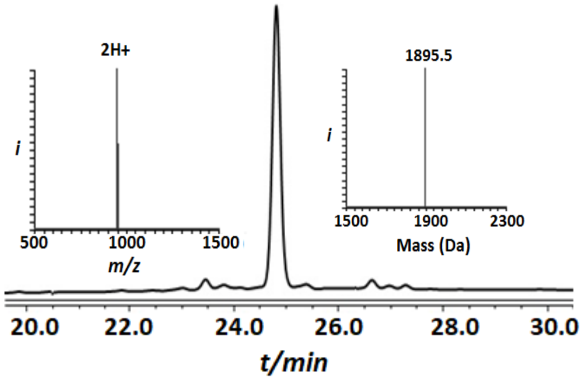

Supplementary Fig. 7 HPLC-MS analysis of cyclic peptide 15, CP1-L1C-CH_3, with the observed mass of 1895.5 ± 0.1 Da (calcd 1895.3 Da, average isotopes).

(ii) Synthesis of cyclic peptide 16, **CP1-L1C-$CH_2C_6H_5$**

Next to Acm removal of 14 (10.0 mg, 5.11X10^{-3} mmol, 1.0 equiv), the aqueous portion was separated and adjusted its pH to 8.0 with NaOH. Subsequently, 500 equiv benzyl bromide ($C_6H_5CH_2Br$) dissolved in 500µl of DMF was added to the reaction mixture at room temperature. The progress of the reaction mixture was monitored by HPLC using a C4 analytical column with a gradient flow of 0-60% B in 30 min. The reaction was completed in 6 h and was purified using a preparative C4 column with a gradient flow of 0-60% in 60 min to give 16, CP1-L1C-$CH_2C_6H_5$ (4.04 mg, 40% yield).

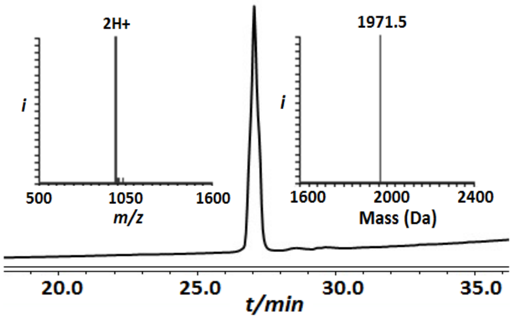

Supplementary Fig. 8 HPLC-MS analysis of cyclic peptide 16, CP1-L1C-$CH_2C_6H_5$, with the observed mass of 1971.5± 0.2 Da (calcd 1971.3 Da, average isotopes).

(iii) Synthesis of cyclic peptide 17, **CP1-L1C-CH_2CONH_2**

Following the Acm removal of 14 (10.0 mg, 5.11X10^{-3} mmol, 1.0 equiv), the aqueous portion was separated and adjusted its pH to 8.0 with NaOH. Next, 500 equiv of 2-iodoacetamide (ICH_2CONH_2) dissolved in 500 µl of DMF was added to the reaction mixture at room temperature. The progress of the reaction mixture was monitored by HPLC using a C4 analytical column with a gradient flow of 0-60% B in 30 min. The reaction was completed in 5 h and was purified using a preparative C4 column with a gradient flow of 0-60% in 60 min to give 17, CP1-L1C-CH_2CONH_2 (4.76 mg, 49% yield).

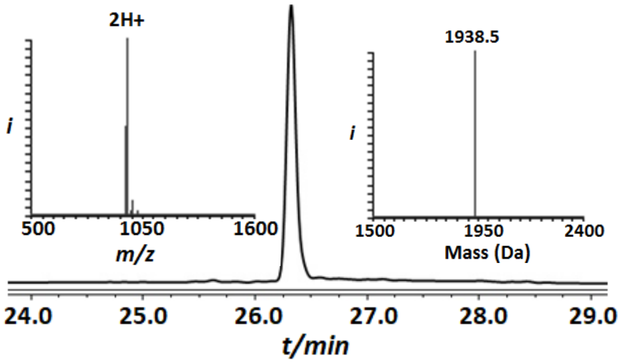

Supplementary Fig. 9 HPLC-MS analysis of cyclic peptide 17, CP1-L1C-CH_2CONH_2, with the observed mass of 1938.5 ± 0.2 Da (calcd 1938.3 Da, average isotopes).

(iv) Synthesis of cyclic peptide 18, CP1-L1C-$CH_2C_{10}H_7$

After Acm removal of 14 (10.0 mg, $5.11X10^{-3}$ mmol, 1.0 equiv), the aqueous portion was separated and adjusted its pH to 8.0 with NaOH. Then, 500 equiv of 2-(bromomethyl)naphthalene ($C_{10}H_7CH_2Br$) dissolved in 500 µl of DMF was added to the reaction mixture at room temperature. The progress of the reaction mixture was monitored by HPLC using a C4 analytical column with a gradient flow of 0-60% B in 30 min. The reaction was completed in 7 h and was purified using a preparative C4 column with a gradient flow of 0-60% in 60 min to give 18, CP1-L1C-$CH_2C_{10}H_7$ (4.14 mg, 40% yield).

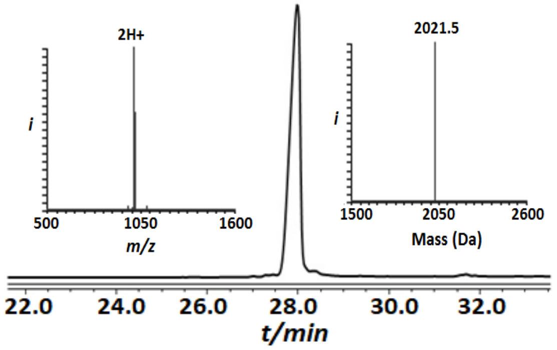

Supplementary Fig. 10 HPLC-MS analysis of cyclic peptide 18, CP1-L1C-$CH_2C_{10}H_7$, with the observed mass of 2021.5± 0.2 Da (calcd 2021.3 Da, average isotopes).

(v) Synthesis of cyclic peptide 19, CP1-L1C-$CH_2C_{10}H_7O_3$

After Acm removal of 14 (10.0 mg, 5.11X10-3 mmol, 1.0 equiv), the aqueous portion was separated and adjusted its pH to 8.0 with NaOH. After quenching and centrifugation (for 5 min at 9169xg) steps of the reaction mixture, separate the supernatant and adjust pH to 8.0 with NaOH. Subsequently, 500 equiv of 4-bromomethyl-7-methoxycoumarin ($BrCH_2C_{10}H_7O_3$) dissolved in 500 µl of DMF was added to the reaction mixture at room temperature. The progress of the reaction mixture was monitored by HPLC using a C4 analytical column with a gradient flow of 0-60% B in 30 min. The reaction was completed in 8 h and was purified using a preparative C4 column with a gradient flow of 0-60% in 60 min to give 19, CP1-L1C-$CH_2C_{10}H_7O_3$ (4.23 mg, 40% yield).

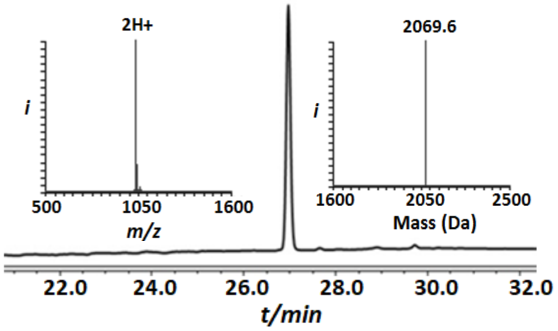

Supplementary Fig. 11 HPLC-MS analysis of cyclic peptide 19, CP1-L1C-$CH_2C_{10}H_7$, with the observed mass of 2069.6 ± 0.2 Da (calcd 2069.3 Da, average isotopes).

(I b) Synthesis of cysteine-arylation derivatives, **20-21**.

(vi) Synthesis of cyclic peptide 20, **CP1-L1C-C_6F_5**

The peptide 2, CP1-L1C (10.0 mg, 5.31X10^{-3} mmol, 1.0 equiv) was dissolved in 2.7 mL of 50 mM Tris base in DMF. Then a solution of hexafluorobenzene (6.15 µl, 10.0 equiv) in 100 µl of DMF was added to the peptide solution.[6] Then vigorously mix the reaction mixture for 30 seconds and kept it at room temperature. The progress of the reaction mixture was monitored by HPLC using a C4 analytical column with a gradient flow of 0-60% B in 30 min. The reaction was completed in 4 h and was purified using a preparative C4 column with a gradient flow of 0-60% in 60 min to give 20, CP1-L1C-C_6F_5 (4.03 mg, 37% yield).

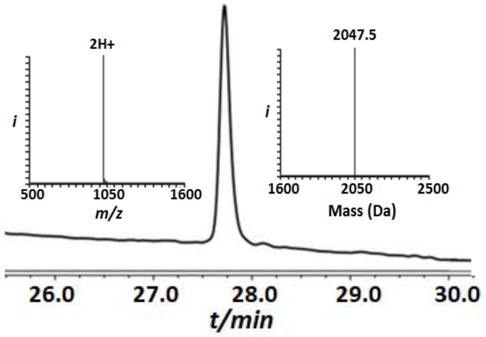

Supplementary Fig. 12 HPLC-MS analysis of cyclic peptide 20, CP1-L1C-C_6F_5, with the observed mass of 2047.5 ± 0.2 Da (calcd 2047.3 Da, average isotopes).

(vii) Synthesis of cyclic peptide 21, CP1-L1C-$C_{10}F_9$

The cyclic peptide 2, CP1-L1C (10.0 mg, 5.31X10^{-3} mmol, 1.0 equiv) was dissolved in 2.7 mL of 50 mM Tris base in DMF. Then a solution of decafluorobiphenyl (5.32 mg, 3.0 equiv) in 100 µl of DMF was added to the peptide solution.[6] Then vigorously mix the reaction mixture for 30 seconds and kept it at room temperature. The progress of the reaction mixture was monitored by HPLC using a C4 analytical column with a gradient flow of 0-60% B in 30 min. The reaction was completed in 5 h and was purified using a preparative C4 column with a gradient flow of 0-60% in 60 min to give 21, CP1-L1C-$C_{10}F_9$ (4.08 mg, 35% yield).

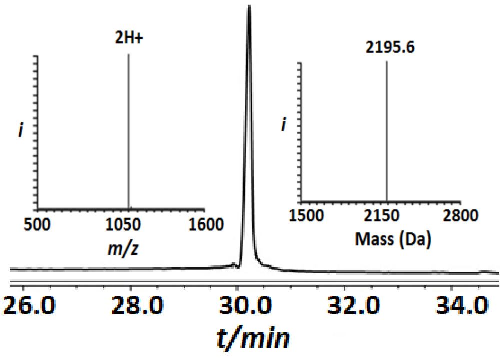

Supplementary Fig. 13 HPLC-MS analysis of cyclic peptide 21, CP1-L1C-$C_{10}F_9$, with the observed mass of 2195.6 ± 0.2 Da (calcd 2195.3 Da, average isotopes).

(II) Binding affinity of cysteine-modified cyclic peptides, 15-21.

The relative binding affinity of the cyclic peptides 15-21 are shown below.

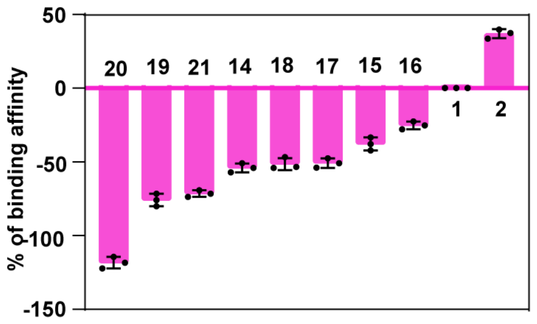

Supplementary Fig. 14 Binding affinity of the derivatives of cyclic peptides 2 to Lys63-linked Di-Ub, no rmalized to the affinity of 1 (CP1). All measurements were performed in n = 3 biologically independent e xperiments. Bar plot represented as mean ± SD. Source data are provided as a source data file.

Synthesis of CP1-FITC and CP1-TAMRA

Fmoc-Cys(Acm) was coupled as the first amino acid for a late-stage modification. After cyclization and Acm removal steps, the peptide with free thiol functional group was dissolved in 6M Gnd·HCl/200 mM phosphate buffer (pH 7.50, 2 mM). Then fluorescein-5-maleimide (2.0 equiv)/tetramethylrhodamie-5-maleimide (TAMRA, 1.50 equiv) dissolved in DMF was added to peptide solution and kept at room temperature under dark conditions. The reaction progress was monitored by HPLC using a C4 analytical column with a gradient flow of 0-60% B in 30 min. The reaction was completed in 2 h and was filtered and purified by injecting into the HPLC using a C4 semi prep-column with a gradient flow of 0-60% in 60 minutes.

a

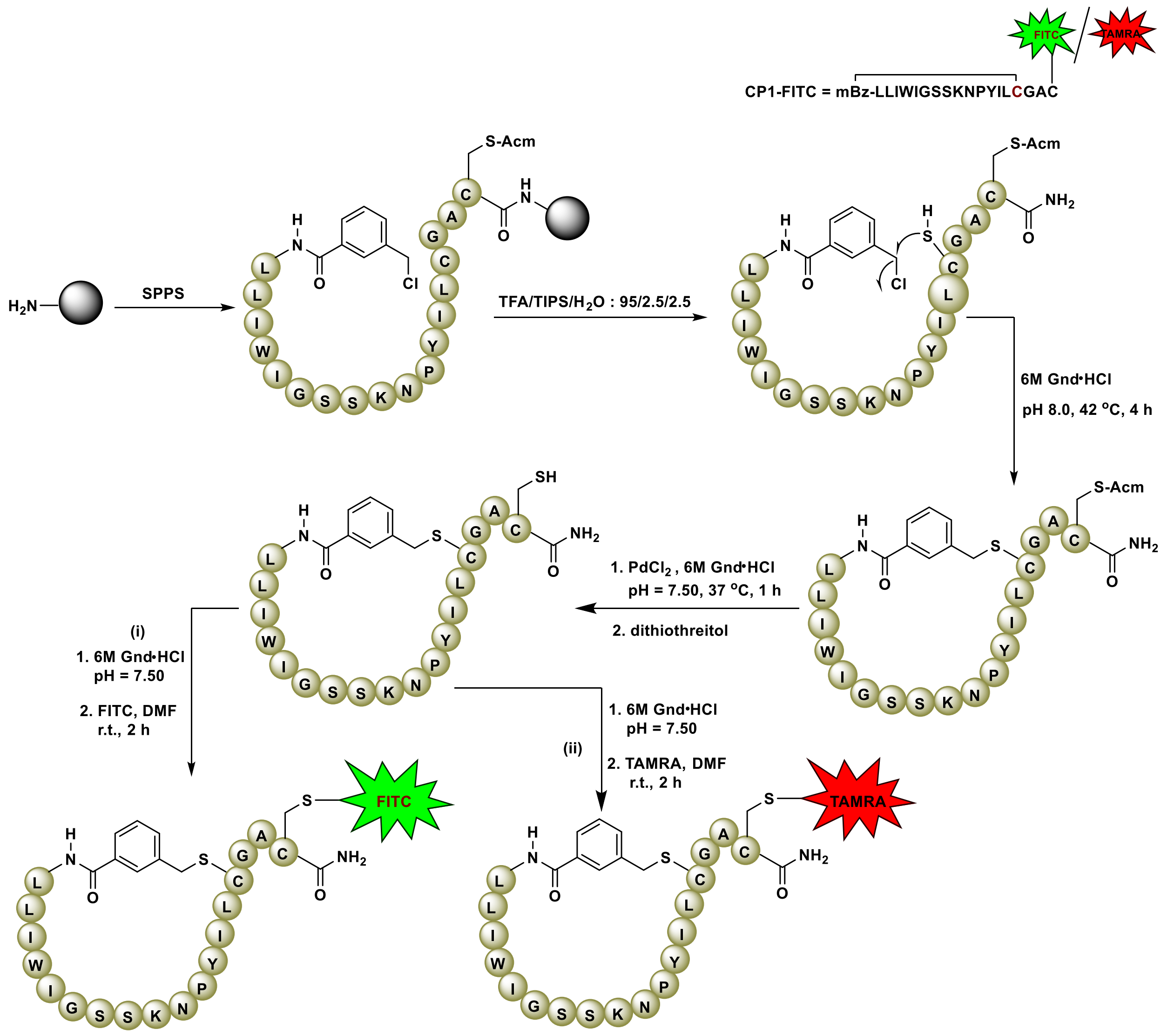

b

(i) CP1-FITC **(ii) CP1-TAMRA**

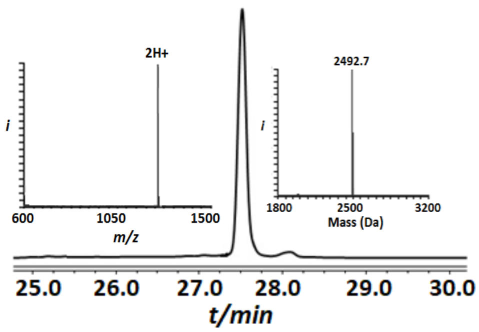

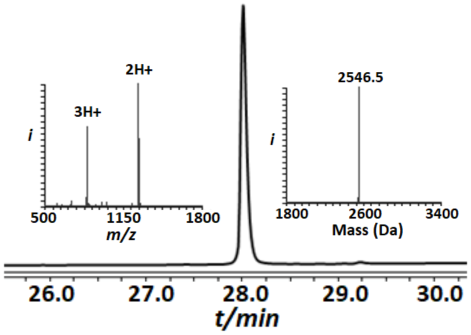

Supplementary Fig. 15 Synthesis of CP1-FITC cyclic peptide. **a** Schematic presentation for the synthesis of CP1-FITC. **b** (i) HPLC-MS analysis of FITC labeled cyclic peptide, CP1-FITC with the observed mass of 2492.7 ± 0.1 Da (calcd 2492.5 Da, average isotopes). (ii) HPLC-MS analysis of TAMRA labeled cyclic peptide, CP1-TAMRA with the observed mass of 2546.5 ± 0.1 Da (calcd 2546.5 Da, average isotopes).

Synthesis of cyclic peptide 23

Fmoc-SPPS was applied for synthesizing the peptide 22. All amino acids were coupled on an automated peptide synthesizer. For synthesizing TAMRA-labeled peptide, cysteine at 1 & 18 positions is orthogonally protected with Acm and -S^{t}Bu respectively for later stage modifications. After coupling 3-(chloromethyl) benzoic acid at N-terminus, the resin was washed with DMF, MeOH, DCM and dried under vacuum. The peptide was cleaved from the resin using the cocktail TFA/H_2O/TIS (95:2.5:2.5) and then precipitation in cold diethyl ether and lyophilization to give 22. The cyclization was performed by dissolving crude peptide 22 (4.0 mM) in 6M Gnd·HCl/200 mM phosphate buffer and adjusted to pH 8.0 with NaOH followed by incubation at 42 °C. The reaction progress was monitored by HPLC using C4 analytical column with a gradient flow of 0-60% B in 30 min, showing reaction completion within 4 h and was purified by HPLC using preparative column C4 with a gradient flow of 0-60% in 60 minutes to give peptide 23 in 43% yield.

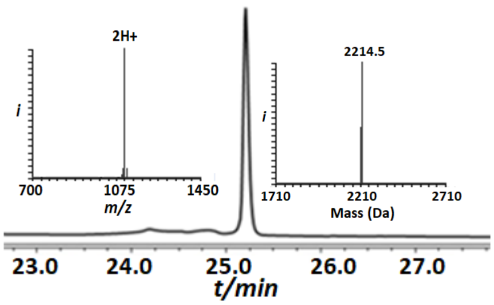

Supplementary Fig. 16 HPLC-MS analysis of cyclic peptide 23 with the observed mass of 2214.5 ± 0.1 Da (calcd 2214.5 Da, average isotopes).

Synthesis of cyclic peptide 24

Peptide 23 (10.0 mg, 4.52X10^{-3} mmol, 1.0 equiv) was dissolved in 6M Gnd·HCl /200mM phosphate buffer (pH 7.50, 2258 µl, 2 mM). To this, a solution of TCEP (50.0 equiv) dissolved in H_2O was added following adjusted pH to 2.50 and incubated at 37 °C.[7] The reaction progress was monitored by HPLC using a C4 analytical column with a gradient flow of 0-60% B in 30 min. The reaction was completed within 4 h and was purified by HPLC using semi-preparative column C4 with a gradient flow of 0-60% in 60 minutes to give peptide 24 (3.84 mg, 40% yield).

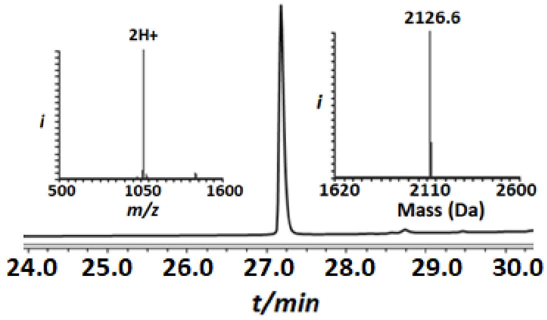

Supplementary Fig. 17 HPLC-MS analysis of cyclic peptide 24 with the observed mass of 2126.6 ± 0.2 Da (calcd 2126.5 Da, average isotopes).

Synthesis of cyclic peptide 25

Peptide 24 (10.0 mg, 4.70X10^{-3} mmol, 1.0 equiv) was dissolved in 6M Gnd·HCl /200mM phosphate buffer (pH 7.50, 2351 µl, 2 mM). To this, add tetramethylrhodamine-5-maleimide (3.40 mg, 1.50 equiv) dissolved in 50 µl of DMF at room temperature under dark conditions. The reaction progress was monitored by HPLC using a C4 analytical column with a gradient flow of 0-60% B in 30 min. The reaction was completed in 2

h and was purified by HPLC using semi-preparative column C4 with a gradient flow of 0-60% in 60 minutes to give peptide 25 (5.15 mg, 42% yield).

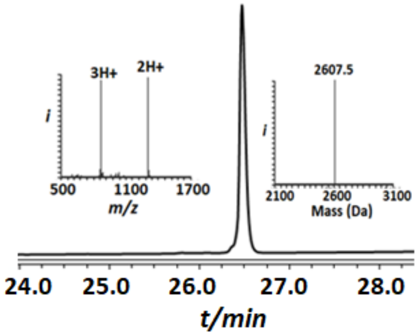

Supplementary Fig. 18 HPLC-MS analysis of cyclic peptide 25 with the observed mass of 2607.5 ± 0.1 Da (calcd 2607.5 Da, average isotopes).

Synthesis of cyclic peptide 2-TAMRA, 26

Cyclic peptide 25 (10.0 mg, $3.84X10^{-3}$ mmol, 1.0 equiv) was dissolved in 6M Gnd·HCl /200mM phosphate buffer (pH 7.50, ~1918 µl, 2 mM). Then, $PdCl_2$ ~1.36 mg, 2.0 equiv) was dissolved in 50 µl of 6M Gnd·HCl/200 mM phosphate buffer (pH 7.50) at 37 °C for 10 min and it was added to the peptide solution following pH adjustment to 1.50 and the reaction was kept at room temperature. The reaction progress was monitored by HPLC using a C4 analytical column with a gradient flow of 0-60% B in 30 min showing reaction completion in 30 minutes. Then the reaction mixture was quenched by adding dithiothreitol, DTT, (8.0 equiv, $3.07X10^{-2}$ mmol) followed by centrifugation (for 5 min at 9169xg) and injection of the supernatant into HPLC using a semi-preparative C4 column with a gradient flow of 0-60% B in 60 min to give peptide 26 (1.85 mg, 19 % yield) with free thiol.

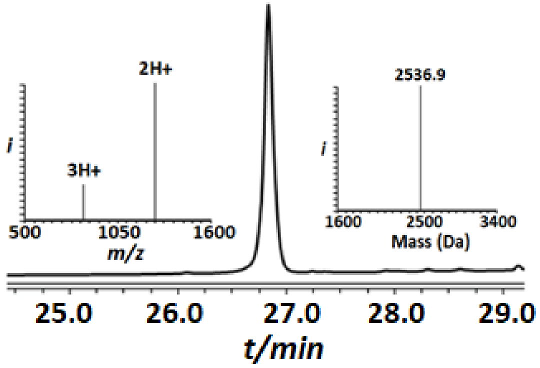

Supplementary Fig. 19 HPLC-MS analysis of cyclic peptide 26 with the observed mass of 2536.9 ± 0.2 Da (calcd 2537.1 Da, average isotopes).

Synthesis of cyclic peptide 27 and 27a

Peptide 26 (10.0 mg, 3.94X10^{-3} mmol, 1.0 equiv) was dissolved in ~ 2 ml of 50 mM Tris-base in DMF. Then a solution of hexafluorobenzene (6.15 µl, 10.0 equiv) in 100 µl of DMF was added to the peptide solution.[6] The reaction mixture was vigorously mixed for 30 seconds and kept at room temperature. The progress of the reaction mixture was monitored by HPLC using a C4 analytical column with a gradient flow of 0-60% B in 30 min, showing reaction completion within 4.5 h. Then the reaction mixture was purified using a preparative C4 column with a gradient flow of 0-60% in 60 min to give 27 (3.30 mg, 31% yield). A similar procedure was used for the synthesis of FITC labeled peptide 27a for cyclic peptide 20.

a

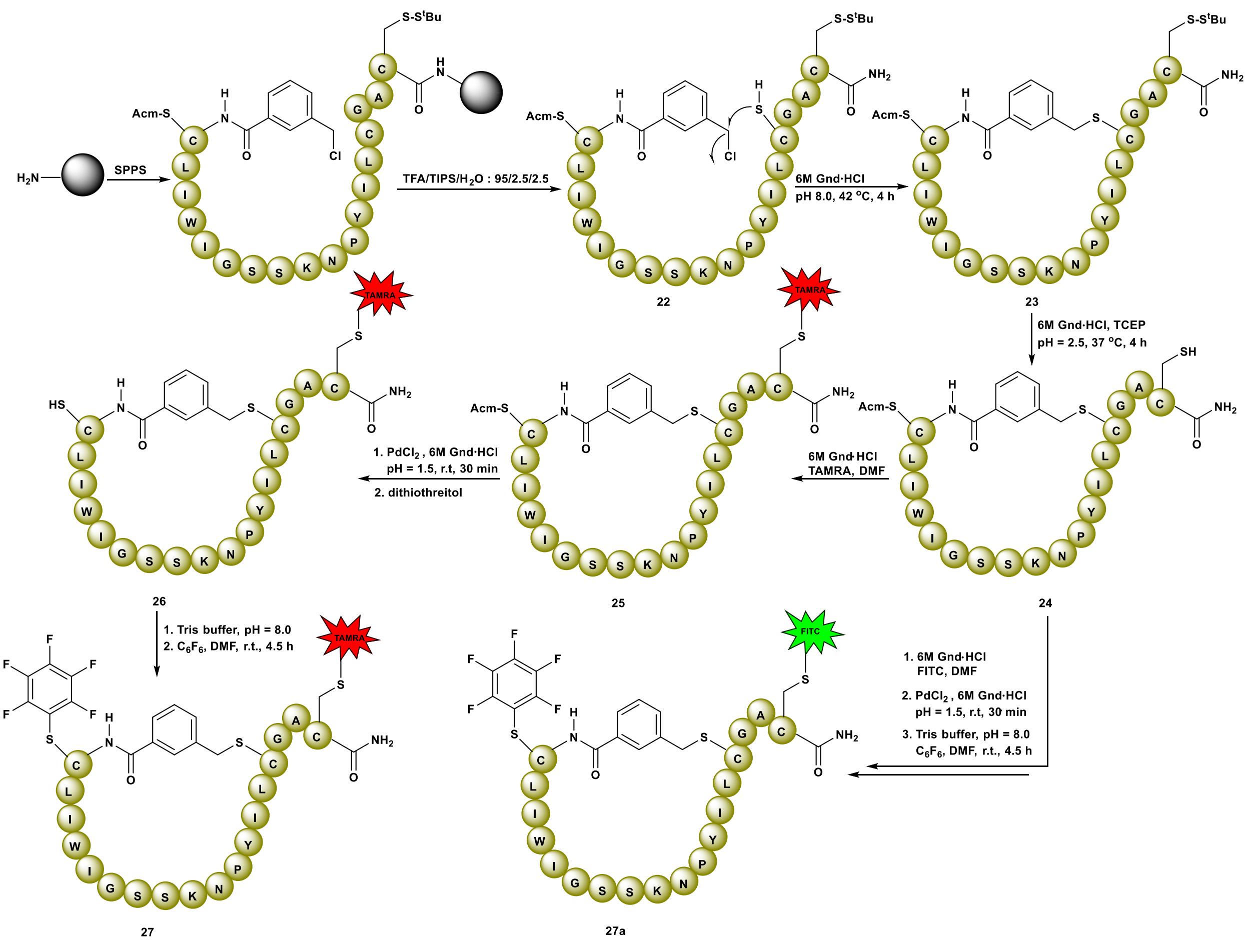

b (i) Compound **26**

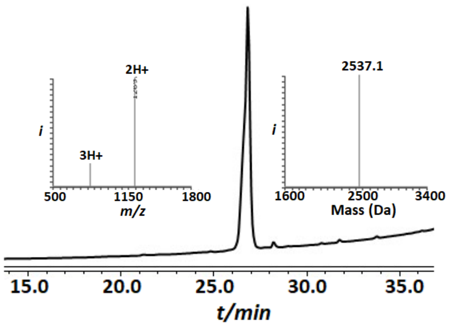

(ii) Compound **27**

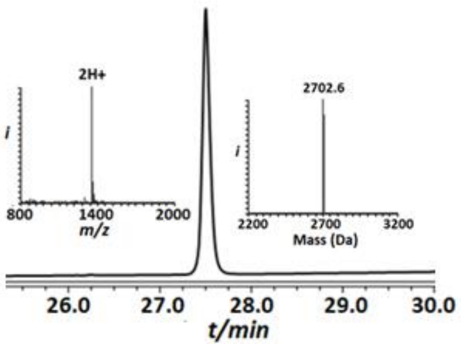

(iii) Compound **27a**

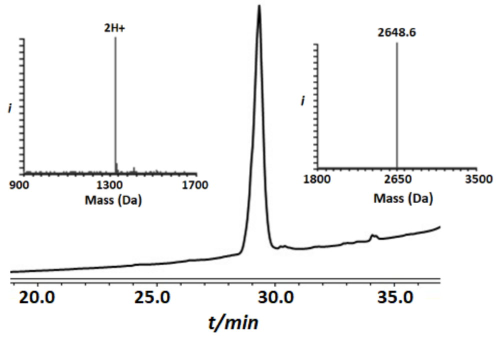

Supplementary Fig. 20 Synthesis of TAMRA/FITC labeled CP1-L1C-C_6F_5 cyclic peptide. **a** Schematic presentation for the synthesis of TAMRA/FITC labeled CP1-L1C-C_6F_5. **b** HPLC-MS analysis of cyclic peptides in which the peak corresponds to (i) 26 with the observed mass of 2537.1 ± 0.2 Da (calcd 2537.2 Da, average isotopes) (ii) 27 with the observed mass of 2702.6 ± 0.2 Da (calcd 2702.5 Da, average isotopes). (iii) 27a with the observed mass of 2648.6 ± 0.2 Da (calcd 2650.4 Da, average isotopes). Where TAMRA = Tetramethylrhodamine-5-maleimide and FITC = Fluorescein-5- maleimide.

Selectivity of peptides 1 and 2 against Ub chains with different linkage

The binding of cyclic peptide 1 against Lys11 and Lys29 linked Di-Ub was examined using surface plasmon resonance (SPR) (Supplementary Fig. 21a,b). The SPR analysis was performed according to the reported protocol.[8] The relative binding affinity of cyclic peptides 1 and 2 for linear Di-Ub as well as Lys6, Lys48 linked Di-Ub and Lys48 linked Tetra-Ub was tested applying our fluorescence-based assay as shown in Supplementary Fig. 21c-f.

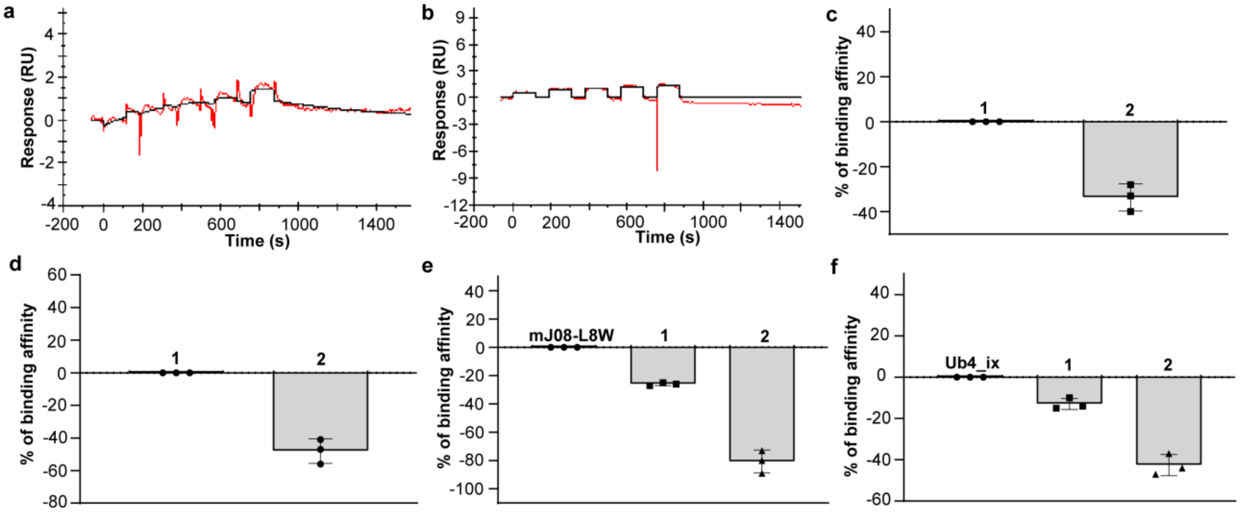

Supplementary Fig. 21 The binding affinity of cyclic peptides 1 and 2 against Ub chains with different linkages and lengths. **a** Binding of cyclic peptide 1 on Lys11 linked Di-Ub, which indicated no binding by SPR. (Red: original trace, Black: fitting curve). **b** Binding of cyclic peptide 1 on Lys29-linked Di-Ub which indicated no binding by SPR. (Red: original trace, Black: fitting curve). **c** Relative binding of cyclic peptides 1 and 2 on linear Di-Ub. Data were plotted as mean ± SD for n = 3 biologically independent experiments. **d** Relative binding of cyclic peptides 1 and 2 on Lys6-linked Di-Ub. Data were plotted as mean ± SD for n = 3 biologically independent experiments. **e** Relative binding of cyclic peptides 1 and 2 on Lys48-linked Di-Ub. Data were plotted as mean ± SD for n = 3 biologically independent experiments. **f** Relative binding of cyclic peptides 1 and 2 on Lys48-linked Tetra-Ub. Data were plotted as mean ± SD for n = 3 biologically independent experiments. All source data are provided as a Source data file.

Synthesis of FITC labeled CP1-L1C -Acm cyclic peptide 28

Cyclic peptide 24 (10.0 mg, $4.70X10^{-3}$ mmol, 1.0 equiv) was dissolved in 6M Gnd·HCl/200 mM phosphate buffer (pH 7.5, 2351 µl, 2 mM) followed by the addition of fluoresceine-5-maleimide (6.02 mg, 3.0 equiv) dissolved in 100 µl of DMF. The reaction was kept at room temperature in dark. The reaction progress was monitored by HPLC using a C4 analytical column with a gradient flow of 0-60% B in 30 min. The reaction

was completed within 2 h and was purified by HPLC using semi-preparative column C4 with a gradient flow of 0-60% in 60 minutes to give peptide 28 in 44% isolated yield (5.28 mg).

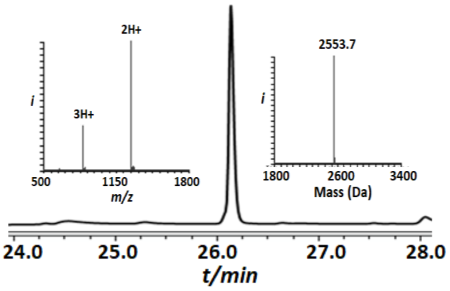

Supplementary Fig. 22 HPLC-MS analysis of cyclic peptide 28 with the observed mass of 2553.7 ± 0.1 Da (calcd 2553.5 Da, average isotopes).

Synthesis of cyclic peptide 2-FITC, 29

Peptide 28 (10.0 mg, $3.92X10^{-3}$ mmol, 1.0 equiv) was dissolved in 6M Gnd·HCl/200 mM phosphate buffer (pH 7.5, 1958 µl, 2 mM). Then, $PdCl_2$ (1.39 mg, 2.0 equiv) was dissolved in 50 µl of 6M Gnd·HCl/200 mM phosphate buffer (pH 7.5) at 37 °C for 10 min and it was added to the peptide solution following adjusted to pH 1.5 and kept the reaction at room temperature. After completion of the reaction, 30 mins, the reaction mixture was quenched with DTT following centrifugation for 5 min at 9169xg and injected supernatant into HPLC using a semi-preparative C4 column with a gradient flow of 0-60% B in 60 min to give peptide 29 (2.04 mg, 21 % yield) with free thiol.

a

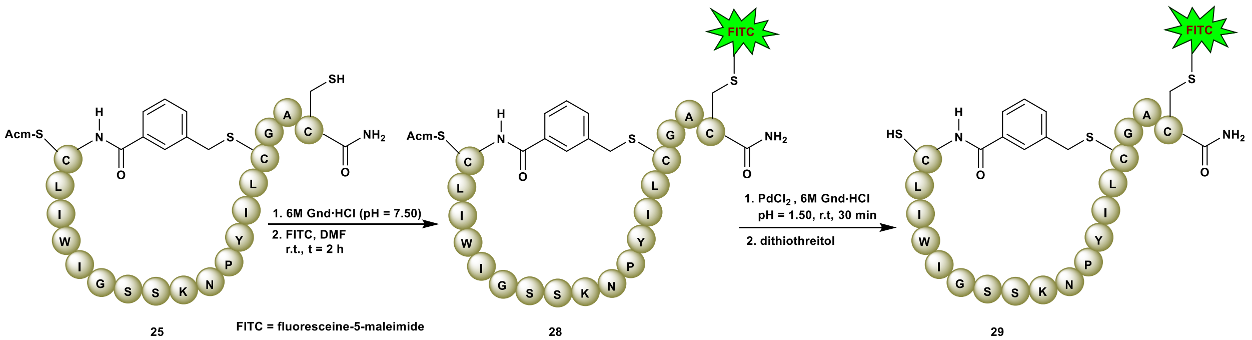

25 28 29

b

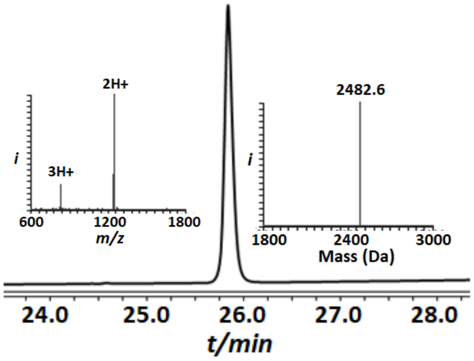

Supplementary Fig. 23 Synthesis of FITC labeled cyclic peptide. **a** Schematic presentation for the synthesis of FITC labeled cyclic peptide 29. **b** HPLC-MS analysis of cyclic peptide 29 with the observed mass of 2482.6 ± 0.1 Da (calcd 2482.5 Da, average isotopes).

Determination of K_D

(i) Determination of K_D of 1 using SPR

The binding of cyclic peptide 1 (CP1) against the target, Lys63 linked Di-Ub was determined using BIACORE T100 instrument (GE Healthcare) equipped with a biotin CAPture kit series S chip. 50 mM HEPES (pH = 7.3, 150 mM NaCl, 0.05% Tween 20, 2.0 mM DTT, 0.2% DMSO) was used as buffer. Biotinylated-Lys63 linked Di-Ub was loaded on the SPR chip. By using various concentrations of peptide 1, the dissociation constant (K_D) was measured to be 16 nM as also described before.[8]

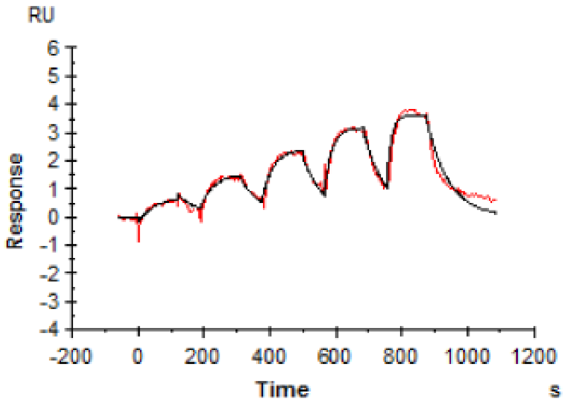

Supplementary Fig. 24 The binding of cyclic peptide 1 against Lys63 linked Di-Ub by SPR.

(ii) Determination of K_D value of CP1-FITC

According to the described procedure in literature,[3] the K_D value of FITC-labeled cyclic peptide 1 (CP1-FITC) was determined. The dissociation constant K_D was calculated as 95.8 ± 2.3 nM.

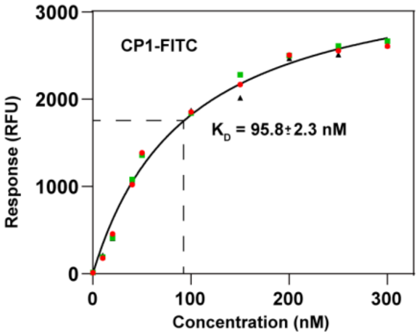

Supplementary Fig. 25 The carve represents the binding of CP1-FITC to Lys63 linked Di-Ub. Using Y = Bmax*X/(K_D + X) formula, the determined K_D value = 95.8 ± 2.3 nM. All measurements were performed in three independent experiments. Bars represent mean values. All source data are provided as a Source data file.

(iii) Determination of K_D value of CP1-TAMRA

The fluorescence values were measured (λ_{ex} = 510 nm and λ_{em} = 565 nm) in a similar way as described for CP1-FITC. The dissociation constant (K_D) value of TAMRA-labeled CP1 (CP1-TAMRA) was calculated as 101.9 ± 3.6 nM. All measurements were performed in triplicates.

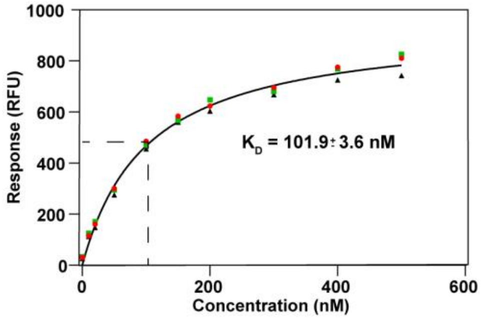

Supplementary Fig. 26 The carve represents the binding of CP1-TAMRA to Lys63 linked Di-Ub. Using Y = Bmax*X/(K_D + X) formula, the determined K_D value = 101.9 ± 3.6 nM. All measurements were performed in triplicates. All measurements were performed in three independent experiments. Bars represent mean values. All source data are provided as a Source data file.

(iv) Determination of K_D value of 2-FITC, 29

The dissociation constant (K_D) value of FITC-labeled cyclic peptide 29 was calculated as 43.2± 4 nM.

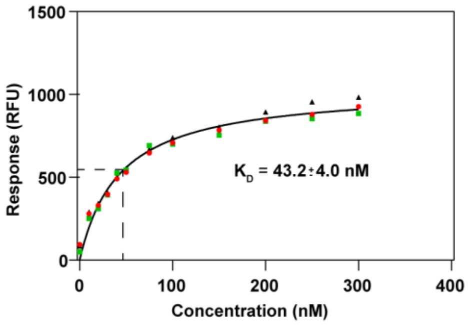

Supplementary Fig. 27 The carve represents the binding of 2-FITC to Lys63 linked Di-Ub. Using Y = Bmax*X/(K_D + X) formula, the determined K_D value = 43.2 ± 4 nM. All measurements were performed in triplicates. All measurements were performed in three independent experiments. Bars represent mean values. All source data are provided as a Source data file.

(v) Determination of K_D value of 2-TAMRA, 26

The fluorescence values were measured (λ_{ex} = 510 nm and λ_{em} = 565 nm) in a similar way as described for CP1-FITC. The dissociation constant (K_D) value of TAMRA-labeled 26 (2-TAMRA) was calculated as 47.4 ± 5.9 nM. All measurements were performed in triplicates.

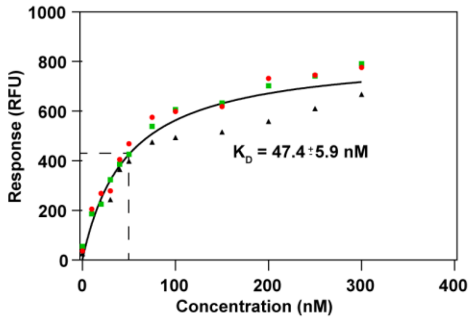

Supplementary Fig. 28 The carve represents the binding of 2-TAMRA to Lys63 linked Di-Ub. Using Y = Bmax*X/(K_D + X) formula, the determined K_D value is 47.4 ± 5.9 nM. All measurements were performed in triplicates. All measurements were performed in three independent experiments. Bars represent mean values. All source data are provided as a Source data file.

(vi) Determination of K_D value of FITC labeled cyclic peptide 27a for cyclic peptide 20

The dissociation constant (K_D) value of FITC-labeled cyclic peptide 27a was calculated as 284.7± 11.9 nM

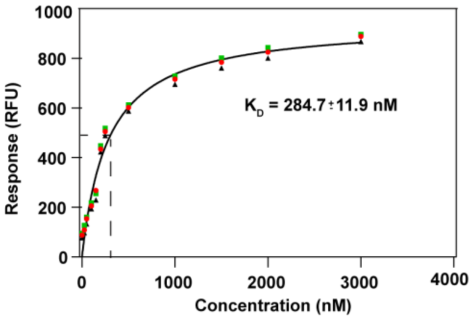

Supplementary Fig. 29 The carve represents the binding of 27a to Lys63 linked Di-Ub. Using Y = Bmax*X/(K_D + X) formula, the determined K_D value = 284.7 ± 11.9 nM. All measurements were performed in triplicates. All measurements were performed in three independent experiments. Bars represent mean values. All source data are provided as a Source data file.

Synthesis of a biotinylated labeled cyclic peptide, 31

Peptide 24 (10.0 mg, $4.70X10^{-3}$ mmol, 1.0 equiv) was dissolved in 6M Gnd·HCl/200 mM phosphate buffer (pH 7.5, 2351 µl, 2 mM). Then, biotinylated-PEG6-maleimide (6.60 mg, $9.40X10^{-3}$ mmol, 2.0 equiv) was dissolved in 50 µl of DMF and was added to the peptide solution at room temperature. The reaction progress was monitored by HPLC using a C4 analytical column with a gradient flow of 0-60% B in 30 min. The reaction was completed in 45 min and was purified by HPLC using semi-preparative column C4 with a gradient flow of 0-60% in 60 minutes to give peptide 30 (9.04 mg, 68% yield). Next, Cys(Acm) deprotection step was performed by applying palladium chemistry. The reaction progress was monitored by HPLC using a C4 analytical column with a gradient flow of 0-60% B in 30 min. The reaction was completed within 30 min, quenched with DTT. Following centrifugation for 5 min at 9169xg, the mixture was injected into HPLC using a semi-preparative C4 column with a gradient flow of 0-60% B in 60 min to give peptide 31 in 22% isolated yield, (1.94 mg).

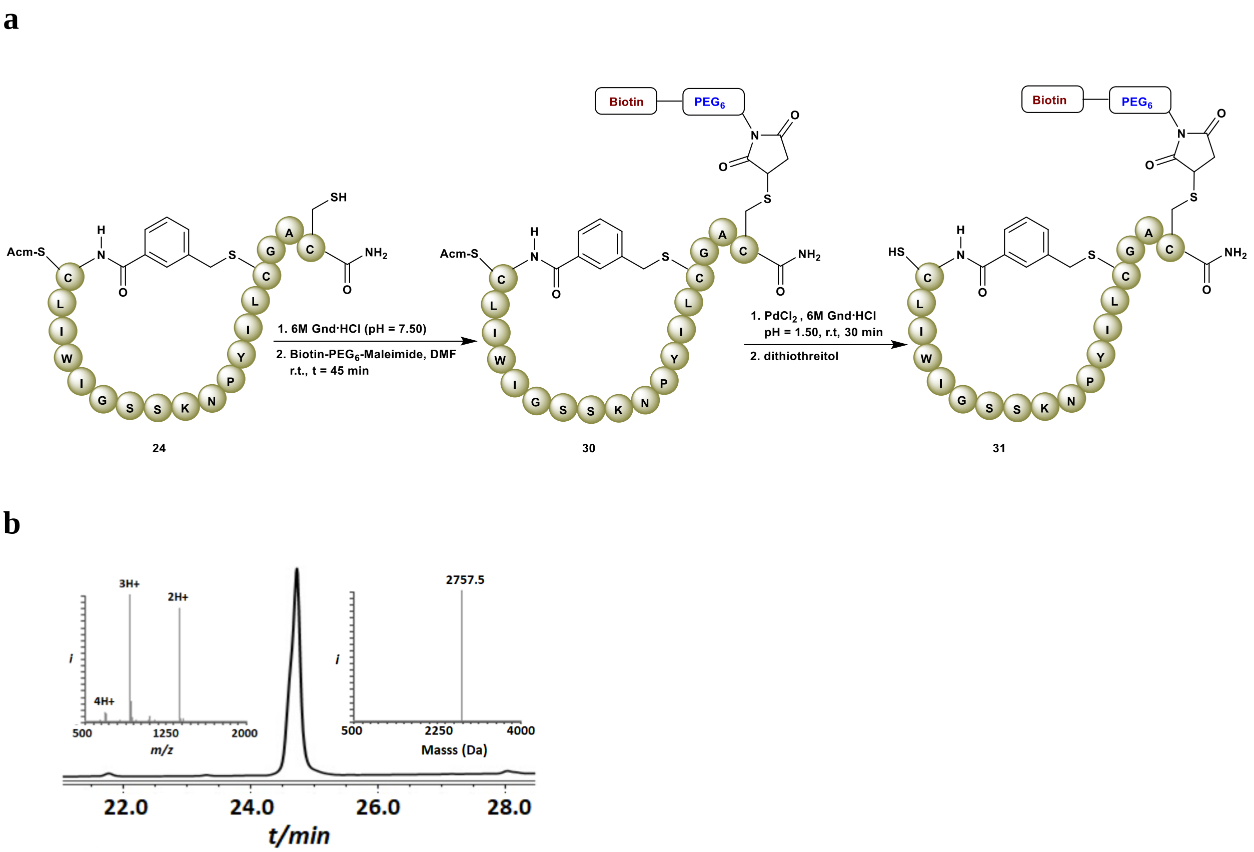

Supplementary Fig. 30 Synthesis of biotinylated cyclic peptide. **a** Schematic presentation for the synthesis of Biotin-PEG$_6$ coupled cyclic peptide 31. **b** HPLC-MS analysis of cyclic peptide 31 with the observed mass of 2757.5± 0.1 Da (calcd 2757.6 Da, average isotopes).

Synthesis and selectivity of a scrambled cyclic peptide, 33

Fmoc-SPPS was applied for synthesizing the scrambled cyclic peptide 33 of cyclic peptide 2. After cleavage from resin and cyclization, purification was performed. For Acm removal,[5] the peptide 32 (10.0 mg, 5.12X10^{-3} mmol, 1.0 equiv) was dissolved in 6M Gnd·HCl/200 mM phosphate buffer (pH 7.5, 2561.5 µl, 2 mM). Then, $PdCl_2$ (9.07 mg, 10.0 equiv) was dissolved in 100 µl of 6M Gnd·HCl/200 mM phosphate buffer (pH 7.5) at 37 °C for 10 min and it was added to the peptide solution. The reaction mixture was incubated at 37 ^{o}C for 1 h. Next, the reaction mixture was quenched with the dithiothreitol, DTT, (40.0 equiv, 2.05X10^{-1} mmol). After centrifugation for 5 min at 9169xg, the supernatant was injected into HPLC using a semi-preparative C4 column with a gradient flow of 0-60% B in 60 min to give free thiol-containing cyclic peptide 33 (4.05 mg, 42 % yield). Next, the relative binding affinity of cyclic peptide 33 for Lys63 linked Di-Ub was compared with specific Lys48 linked Di-Ub binder, mJ08-L8W.

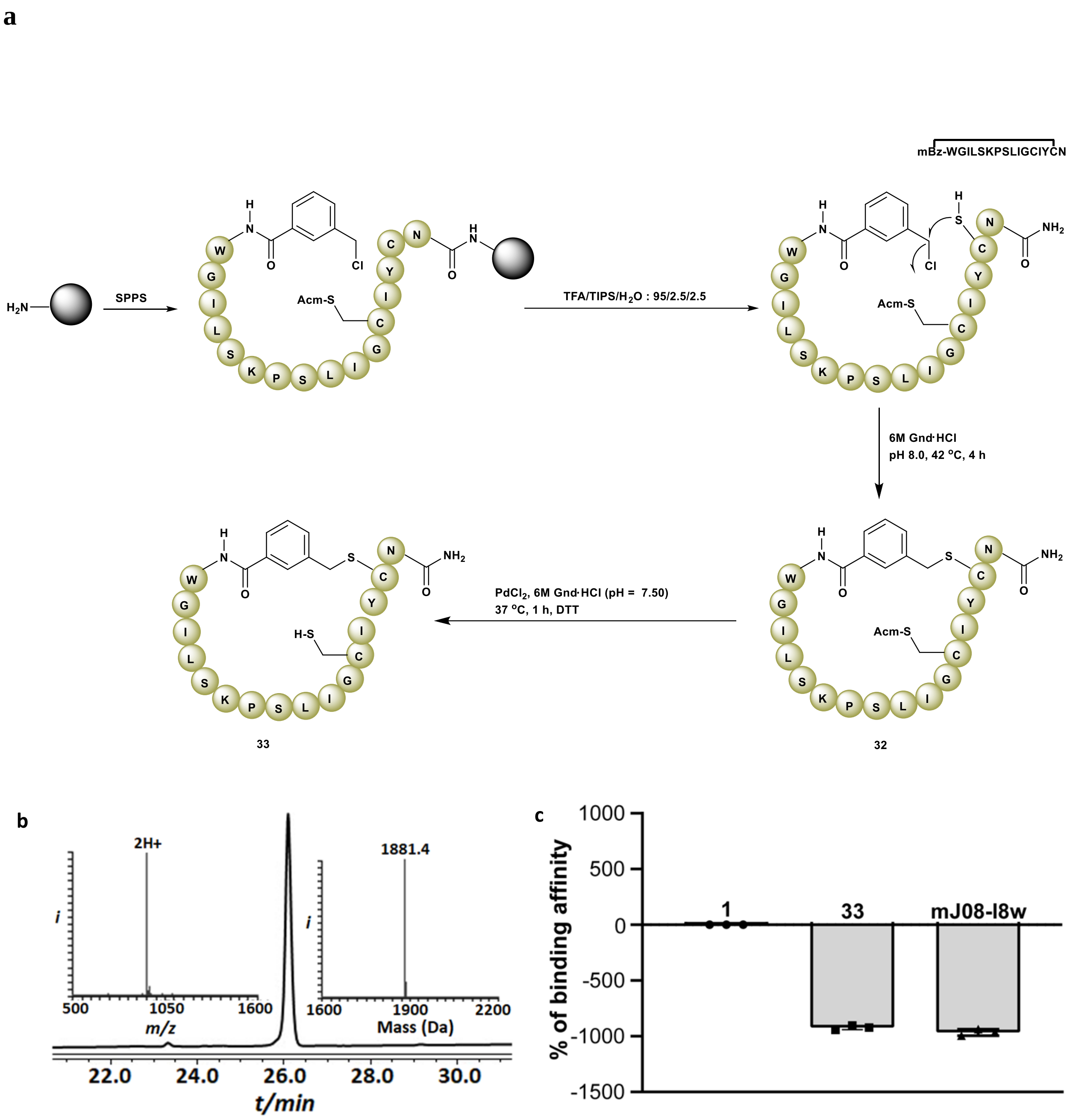

Supplementary Fig. 31 Synthesis of scrambled cyclic peptide 33. **a** Schematic presentation for the synthesis of cyclic peptide 33. **b** HPLC-MS analysis of cyclic peptide 33 with the observed mass of 1881.4 ± 0.1 Da (calcd 1881.3 Da, average isotopes). **c** Relative binding of cyclic peptide 33 and mJ08-L8W on Lys63 linked Di-Ub. All measurements were performed in three independent experiments. Data were plotted as mean ± SD. All source data are provided as a Source data file.

Synthesis of a biotinylated labeled scrambled cyclic peptide, 38

A similar Fmoc-SPPS procedure was applied for synthesizing the peptide 34. For synthesizing biotinylated peptides, cysteine at 1 & 18 positions is orthogonally protected with Acm and -S^{t}Bu respectively for later stage modifications. After coupling 3-(chloromethyl) benzoic acid at N-terminus, the resin was washed with DMF, MeOH, DCM and dried under vacuum. The peptide was cleaved from the resin using the cocktail TFA/H_2O/TIS (95:2.5:2.5) and then precipitation in cold diethyl ether and lyophilization to give 34. The cyclization was performed by dissolving crude peptide 34 (4.0 mM) in 6M Gnd·HCl/200 mM phosphate buffer and adjusted to pH 8.0 with NaOH followed by incubation at 42 °C. The reaction progress was monitored by HPLC using C4 analytical column with a gradient flow of 0-60% B in 30 min, showing reaction completion within 4 h, and was purified by HPLC using preparative column C4 with a gradient flow of 0-60% in 60 minutes to give peptide 35 in 40% yield. S^{t}Bu deprotection was performed using TCEP for the preparation of peptide 36. Then, biotinylated cyclic peptide 37 was prepared. Next, palladium chemistry was applied for Cys(Acm) deprotection. The reaction progress was monitored by HPLC using a C4 analytical column with a gradient flow of 0-60% B in 30 min. The reaction was completed within 30 min, quenched with DTT. Following centrifugation for 5 min at 9169xg, the mixture was injected into HPLC using a semi-preparative C4 column with a gradient flow of 0-60% B in 60 min to give peptide 38 in 20% isolated yield.

a

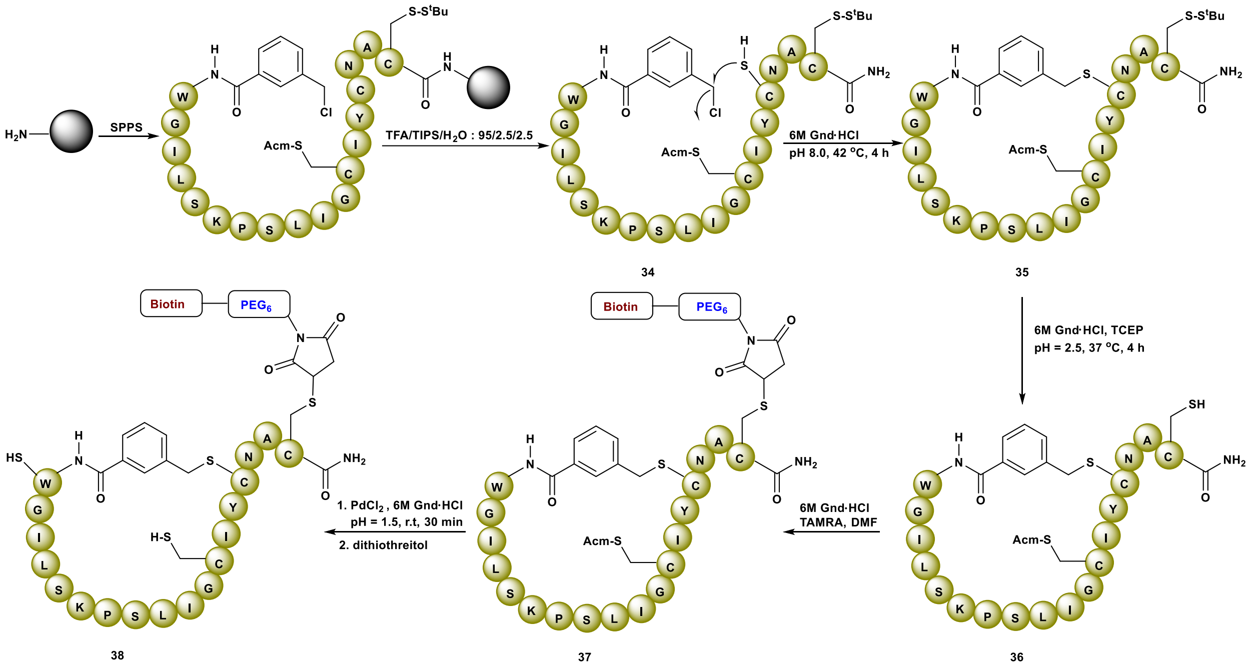

b

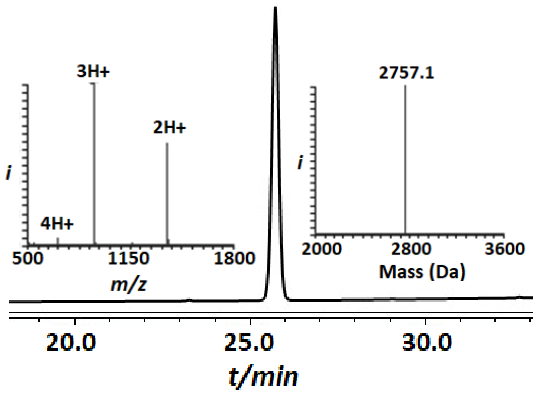

Supplementary Fig. 32 Synthesis of Biotin-PEG6 coupled scrambled cyclic peptide 38. **a** Schematic presentation for the synthesis of biotinylated cyclic peptide 38. **b** HPLC-MS analysis of cyclic peptide 38 with the observed mass of 2757.1 ± 0.1 Da (calcd 2757.6 Da, average isotopes).

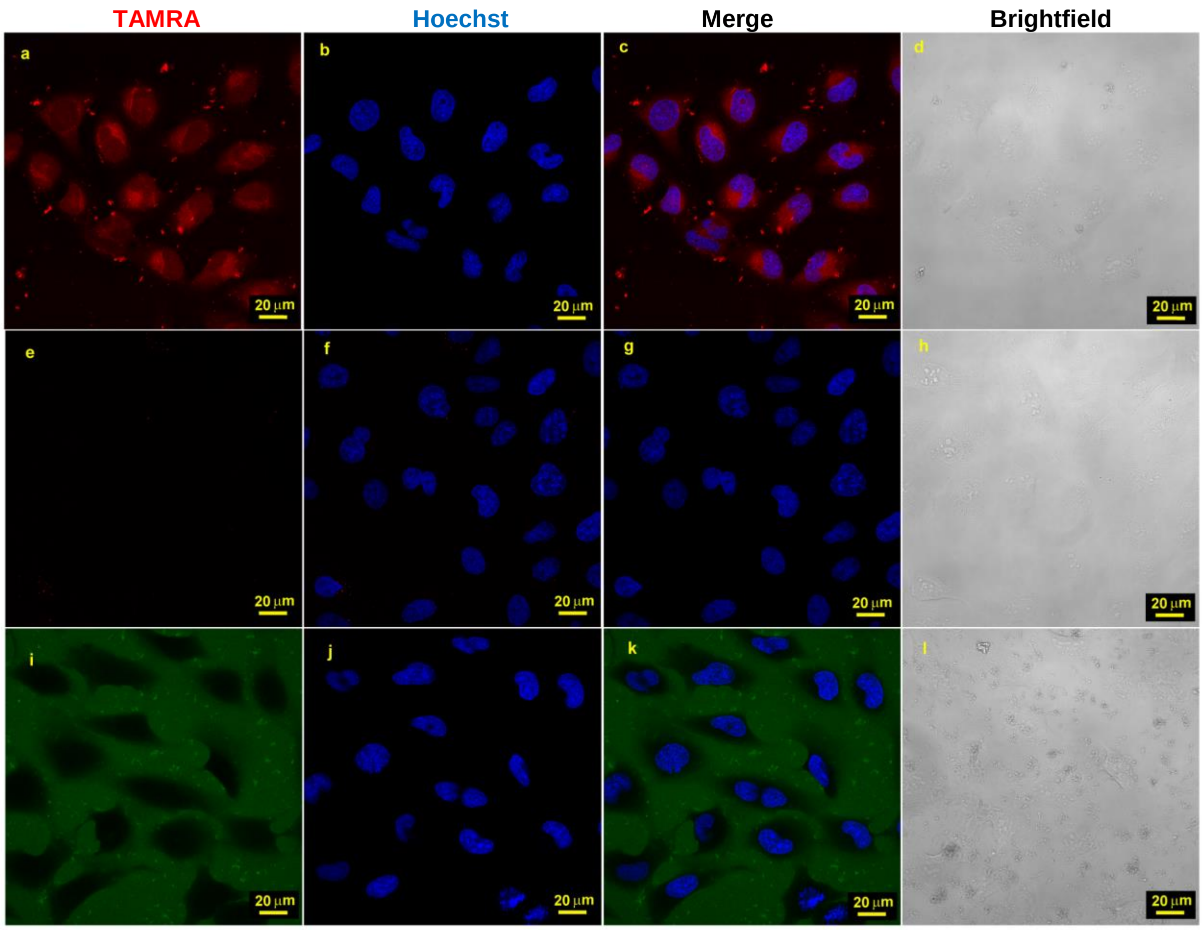

Supplementary Fig. 33 Representative confocal images of delivery of cyclic peptide 26 (2-TAMRA) and 29 (2-FITC) to live U2OS cells. CLSM images of 26 (**a-d**), DMSO (**e-h**), and 29 (**i-l**) treated cells; **a,e** (TAMRA, red channel). **i** (FITC, green channel). **b, f, j** Hoechst (blue channel). **c,g** TAMRA and Hoechst channels combined. **k** FITC and Hoechst channels combined. **d,h,l** Bright field channel. Images representative of n = 2 biologically independent experiments. Scale bars 20 µm.

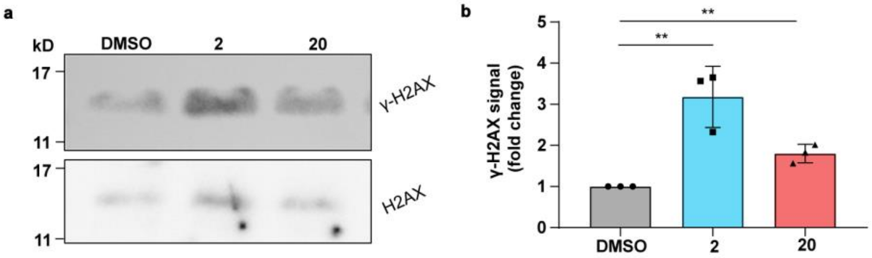

Supplementary Fig. 34 a Western blot analysis of lysates from HeLa cells treated with 2 and 20 (upper panels). H2AX was used as a loading control (lower panels) which was performed in a different blot. Representative of n = 3 biologically independent experiments. **b** Quantified relative γ-H2AX signals (**a**) for n = 3 independent experiments. Data are presented as mean values ± SD. (Two-tailed, unpaired t-test **p [DMSO and 2] = 0.0071 and **p [DMSO and 20] = 0.0035). (DMSO: gray, 2: skyblue, and 20: salmon). Source data are provided as a Source data file.

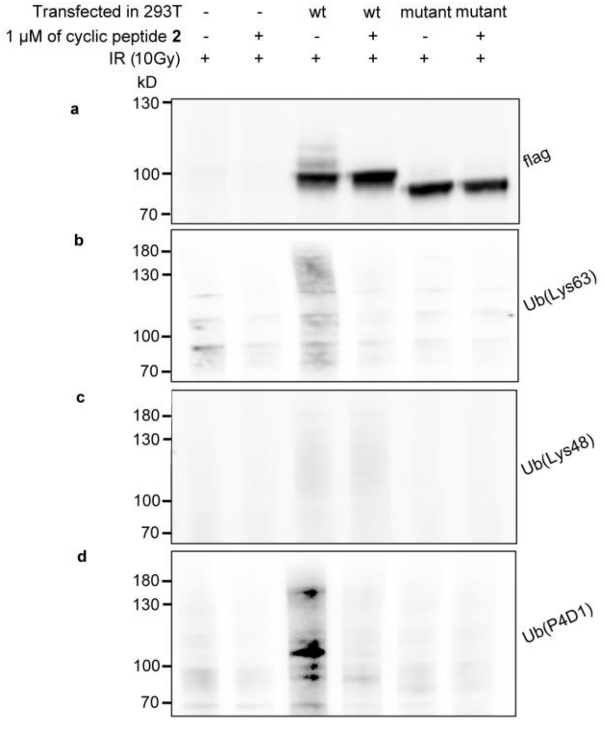

Supplementary Fig. 35 Western blot analysis of protein lysates using antibody for **a** flag, **b** ubiquitin Lys63-specific, **c** ubiquitin Lys48-specific, and **d** non-specific ubiquitin(P4D1) after cell lysates treated with DMSO or 2 and immunoprecipitated from anti-flag. Human Embryonic Kidney 293T cells were overexpressed with RNF168 WT or its dead mutant. Representative of n = 2 biologically independent experiments. Representative full blot images provided as source data file.

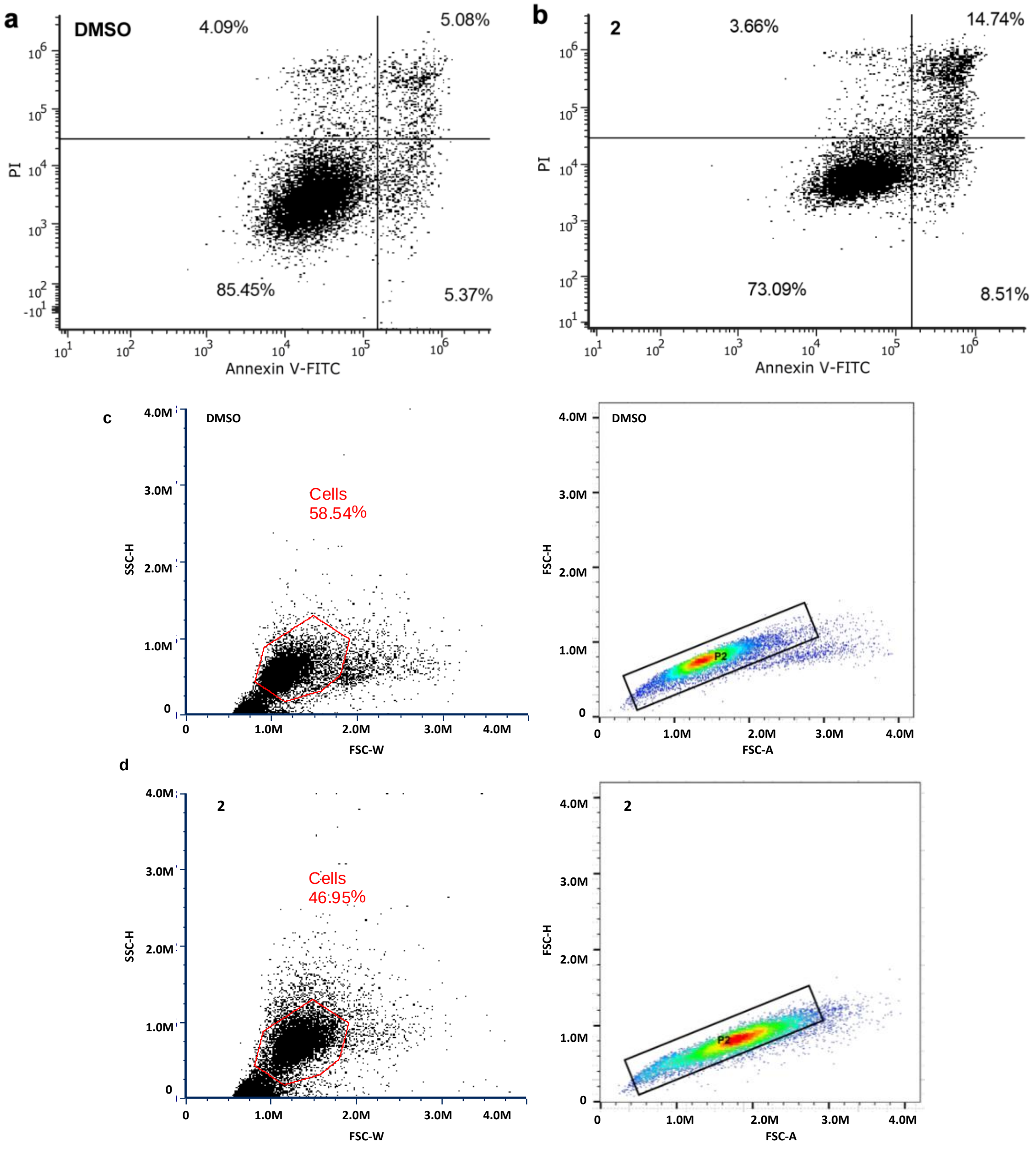

Supplementary Fig. 36 Cells were double-stained with Annexin V-FITC and PI, subsequently analyzed by CYTEK Aurora flow cytometer. **a-b** HeLa cells treated with DMSO (**a**) and cyclic peptide 2 (**b**) for 96 h, and representative dot plot of n = 2 biologically independent experiments were shown here (>20,000 cells each condition). **c-d** Gating plots were shown for cells treated with DMSO (**c**) and cyclic peptide 2 (**d**).

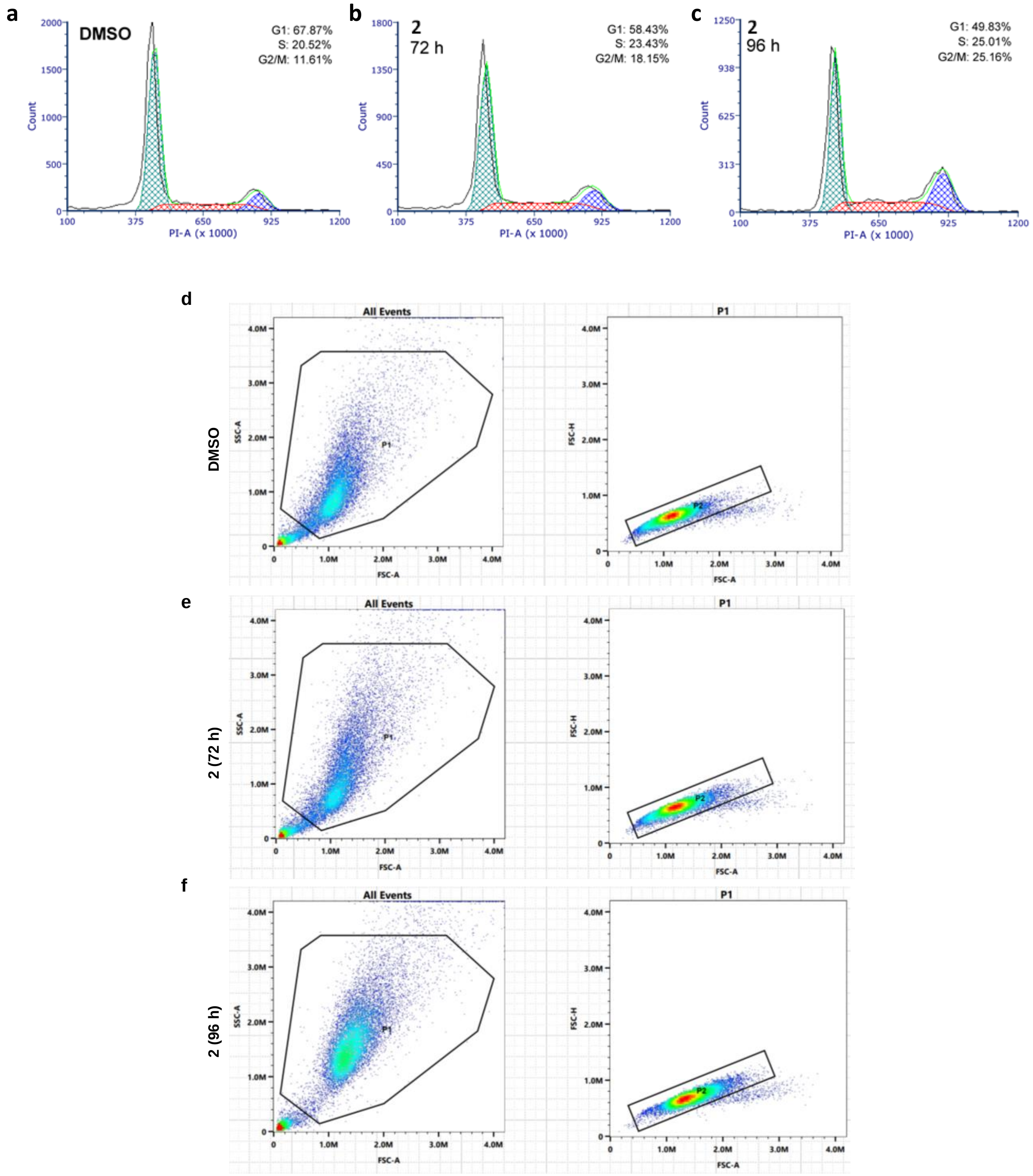

Supplementary Fig. 37 a-c Cell cycle distribution of HeLa cells treated with DMSO (**a**), cyclic peptide 2 for 72 (**b**) and cyclic peptide 2 for 96 h (**c**). The representative heat plots showed a relative population of cells at G1, S, and G2/M phases in green, red, and blue boundaries, respectively (>15,000 cells each condition). Representative histograms of n = 2 independent experiments. **d-f** Gating plots were shown for cells treated with DMSO (**d**), cyclic peptide 2 for 72 (**e**), and cyclic peptide 2 for 96 h (**f**).

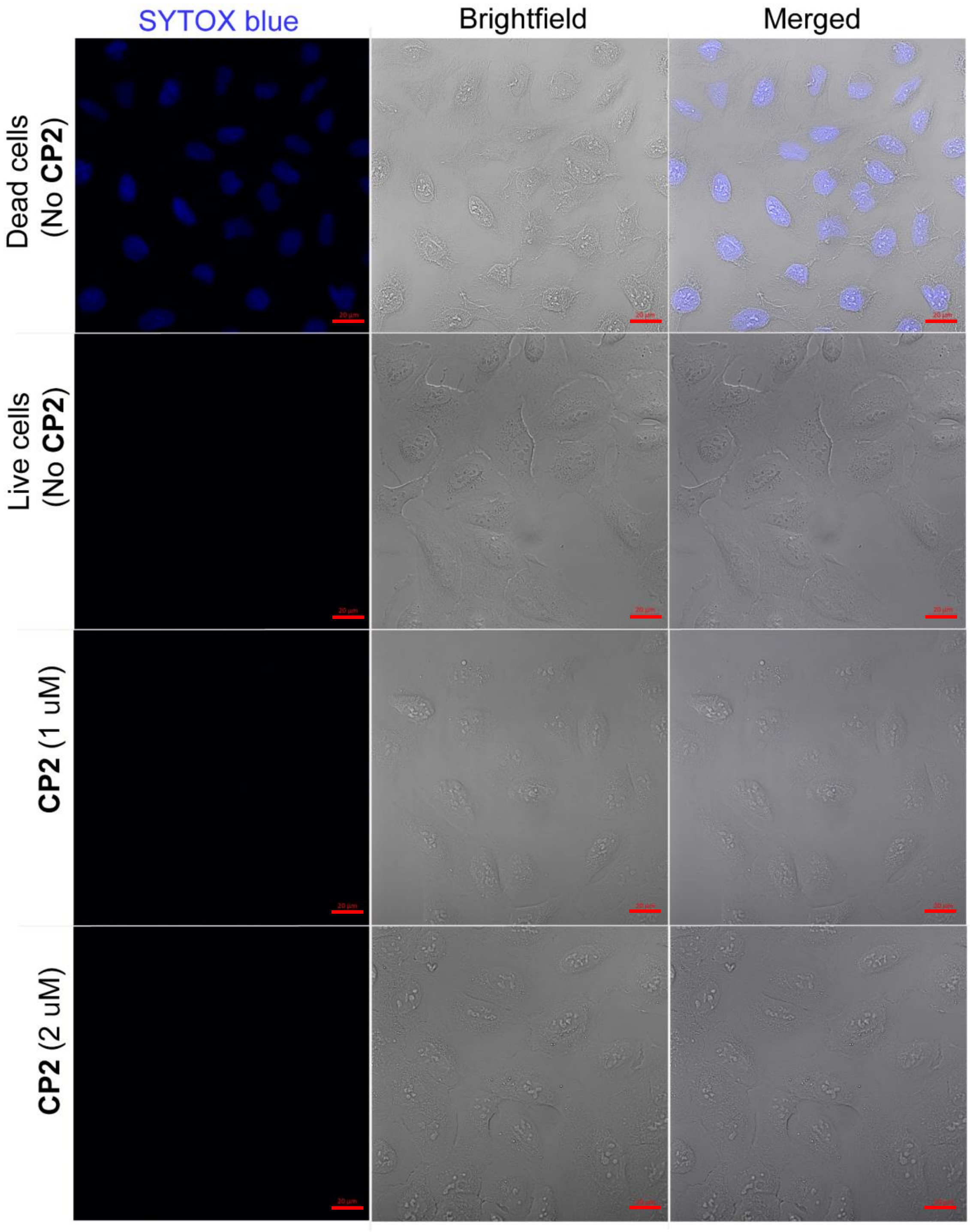

Supplementary Fig. 38 Live U2OS cells treated at various concentrations of cyclic peptide 2 with SYTOX Blue stain. Representative images of n = 2 biologically independent experiments are shown in the first column, for the blue channel; the second column: brightfield; and the third column: merge channel for blue and brightfield; Scale bars 20 μm.

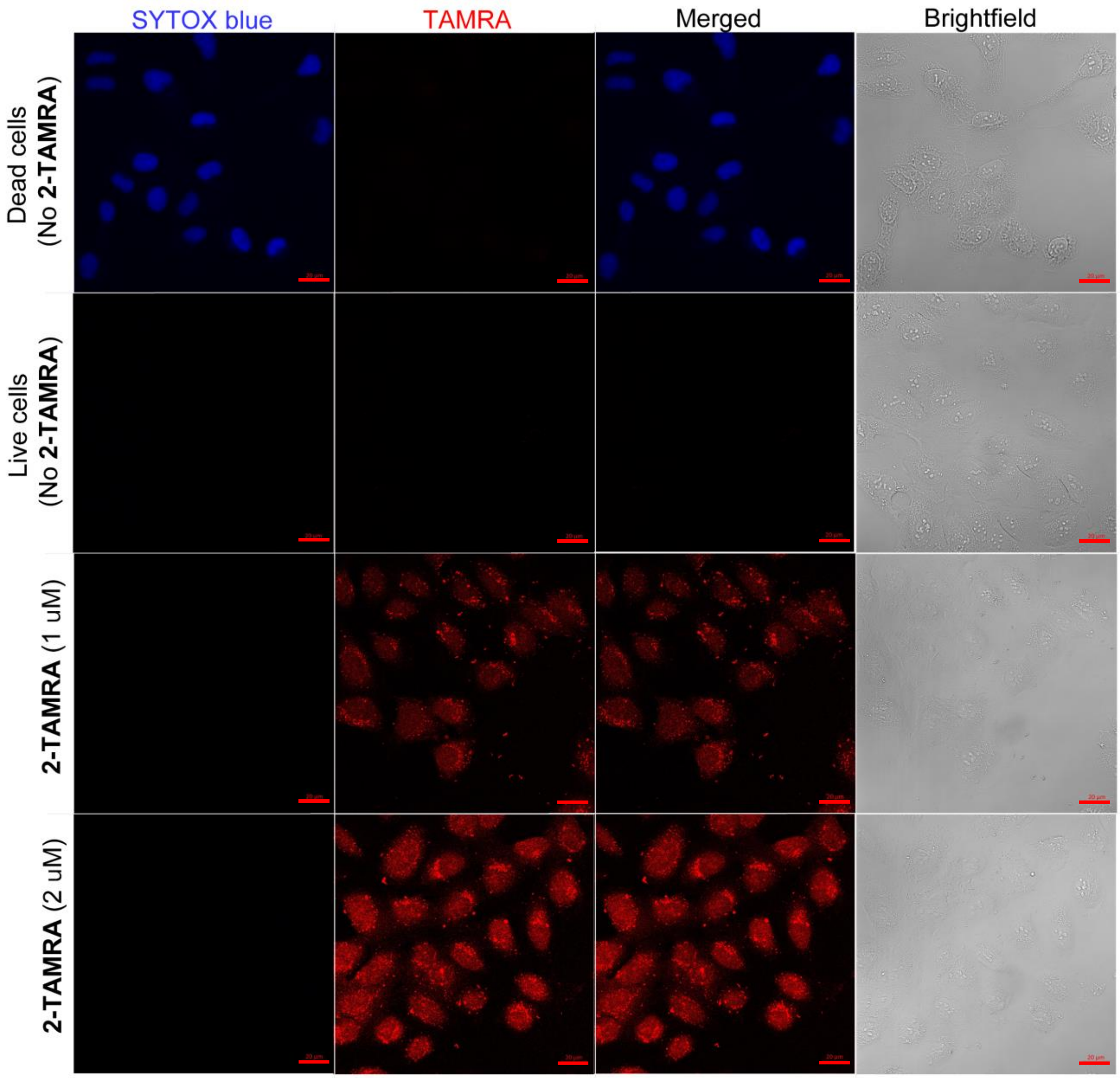

Supplementary Fig. 39 Live U2OS cells treated at various concentrations of cyclic peptide 2 and 2-TAMRA (26) with SYTOX Blue stain. Representative images of n = 2 biologically independent experiments are shown in the first column, for the blue channel; the second column: TAMRA (red channel); the third column: merge channel for blue and red; and the fourth column: brightfield. Scale bars 20 μm.

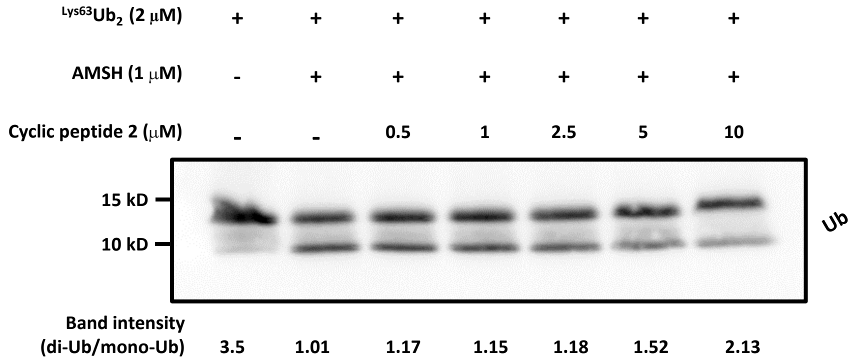

Supplementary Fig. 40 Lys63-linked deubiquitylating (DUB) inhibition activity of the cyclic peptide 2. Band intensities were calculated using FiJi software (ImageJ, open version). Representative image of n = 2 biologically independent experiments. Representative full blot image provided as source data file.

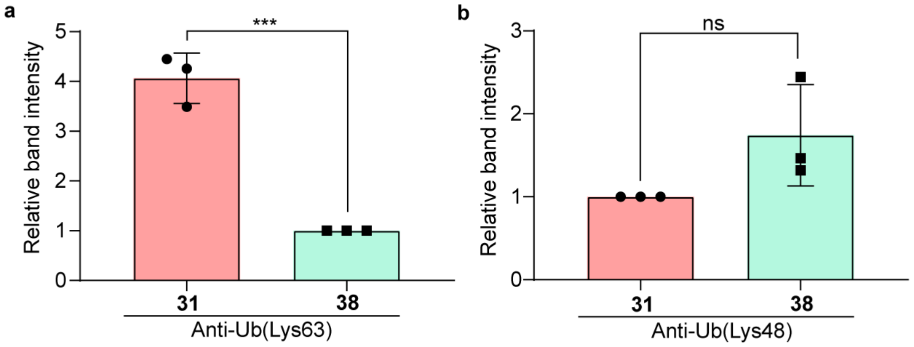

Supplementary Fig. 41 The pulled-down Ub chains (Lys63, and Lys48-linked) by Biotin-conjugated cyclic peptide 2 shown in Fig. 5b were quantified using FiJi (ImageJ, open access). Relative band intensity for the different blots for **a** (Lys63-specific)ubiquitin antibody and **b** (Lys63-specific)ubiquitin antibody were plotted as mean of n = 3 biologically independent experiments and bar graph represents mean ± SD and statistical significance shown as two-tailed, unpaired t-test ***P = 0.0005 and ns is non-significant. Source data are provided as a Source data file.

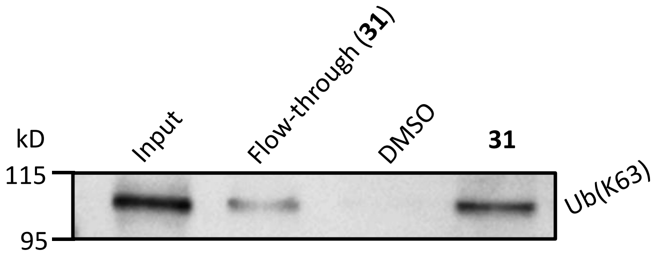

Supplementary Fig. 42 Ub chains were pulled down with and without cyclic peptides 31 and detected by antibodies for Lys63-linked Ub chains in western blot analysis. Representative of n = 2 biologically independent experiments. Representative full blot image provided as source data file.

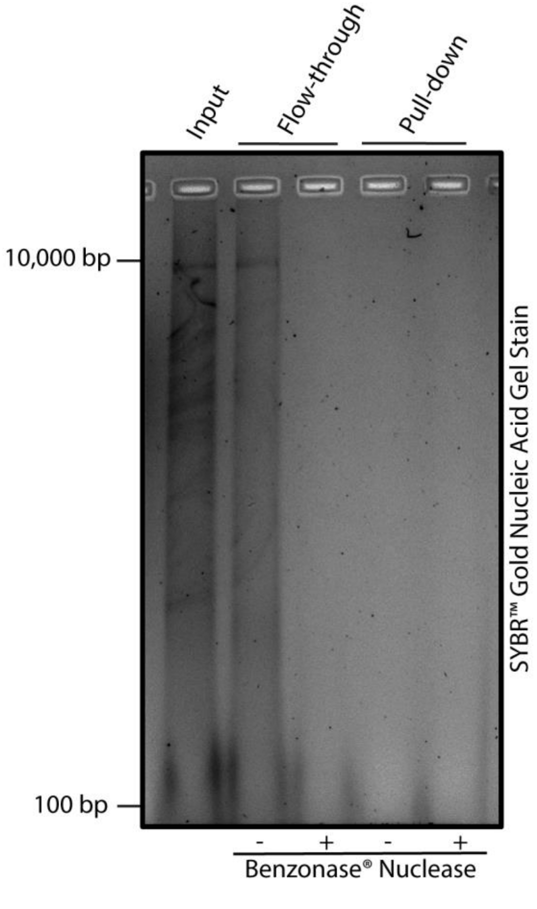

Supplementary Fig. 43 Agarose gel electrophoresis and subsequent staining with the SYBR® Gold of the pulled down components. Pull-down from cell lysate by 31 pretreated with and without Benzonase Nuclease. Representative of n = 2 biologically independent experiments.

Supplementary Table 2 Various enriched terms by 31, classified based on their specific role in cellular processes or functions

Cellular process or functions	Enriched terms of proteins (Identified by their gene names)
DSB repair	TRIP13, XRCC3, PMS2, MLH1, SMARCAD1, FANCD2, RAD50, RAD51, ATM, HUS1, APBB1, TONSL, MMS19, WDR48
Specific Lys63-linked Ub binding	UBR5, PCNA, BABAM1, and PSMD14
DSBs responsive and works RNF8, RNF168 dependent manner	VCP
Phosphorylates 'Ser139' of histone variant H2AX and regulating DSB response mechanism	PRKDC

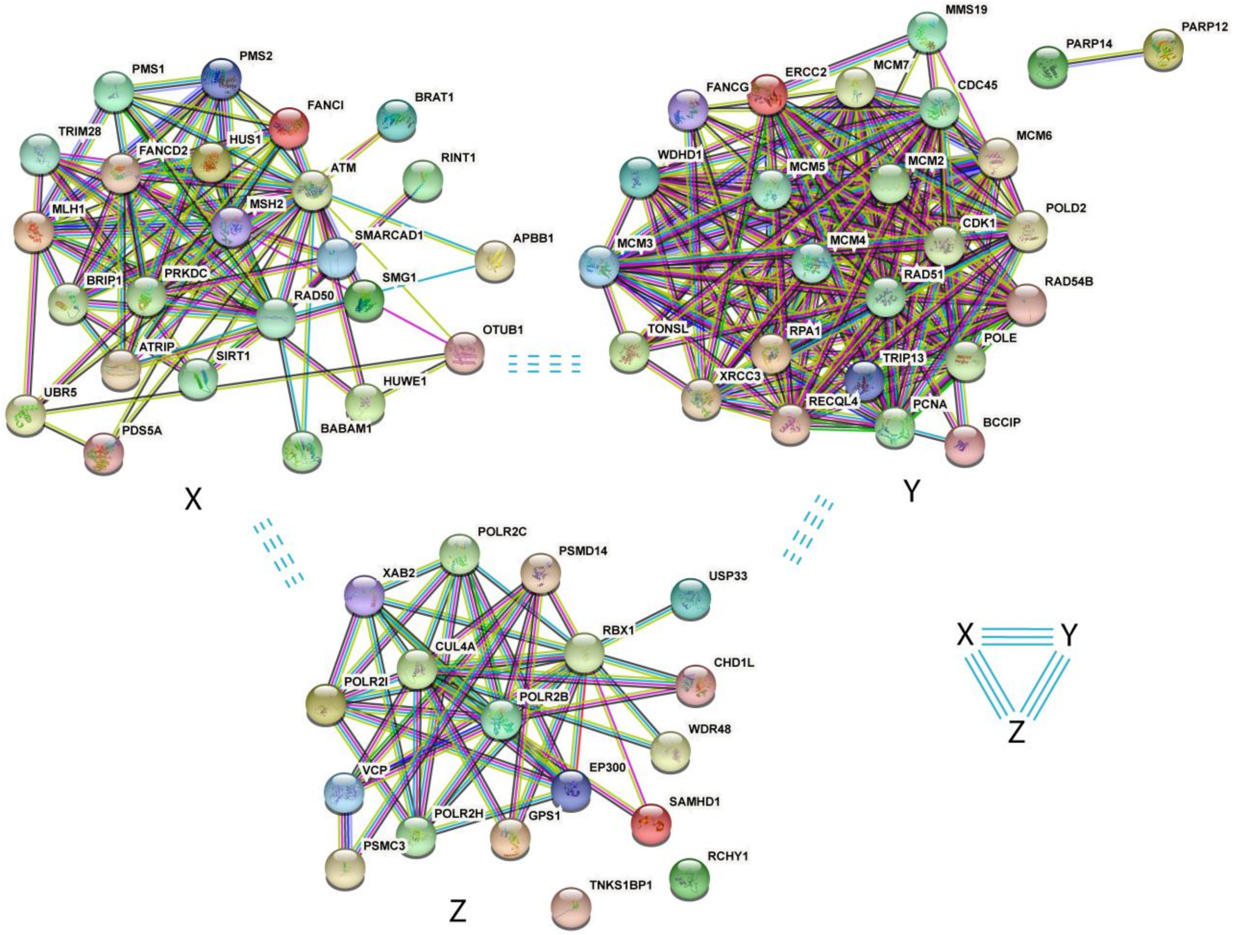

Supplementary Fig. 44 Proteomics analysis after pull-down from streptavidin beads of Lys63-linked Ub modified protein components in U2OS cell lysate. STRING network of DDR proteins (identified by their

gene names) enriched by 31. Data were shown for at least 3-fold enrichment compared to scramble control 38. The experiment was performed in triplicate.

Supplementary References

1. Kumar, K. S. A., Spasser, L., Erlich, L. A. & Bavikar, S. N. Total chemical synthesis of Di-ubiquitin chains. *Angew. Chemie - Int. Ed.* **49**, 9126–9131 (2010).

2. Kumar, K. S. A. *et al.* Total chemical synthesis of a 304 amino acid K48-linked tetraubiquitin protein. *Angew. Chemie - Int. Ed.* **50**, 6137–6141 (2011).

3. Vamisetti, G. B., Meledin, R., Nawatha, M., Suga, H. & Brik, A. The development of a fluorescence-based competitive assay enabled the discovery of dimeric cyclic peptide modulators of ubiquitin chains. *Angew. Chemie - Int. Ed.* **60**, 7018–7023 (2021).

4. Huang, Y. *et al.* Affinity maturation of macrocyclic peptide modulators of Lys48-linked diubiquitin by a twofold strategy. *Chem. - A Eur. J.* **26**, 8022–8027 (2020).

5. Maity, S. K., Jbara, M., Laps, S. & Brik, A. Efficient palladium-assisted one-pot deprotection of (acetamidomethyl)cysteine following native chemical ligation and/or desulfurization to expedite chemical protein synthesis. *Angew. Chemie - Int. Ed.* **55**, 8108–8112 (2016).

6. Spokoyny, A. M. *et al.* A perfluoroaryl-cysteine SNAr chemistry approach to unprotected peptide stapling. *J. Am. Chem. Soc.* **135**, 5946–5949 (2013).

7. Comasseto, J. V. & Guarezemini, A. S. Selective reduction of disulfides by tris(2-carboxyethyl)phosphine. *J. Org. Chem.* 2648–2650 (1991).

8. Nawatha, M. *et al.* De novo macrocyclic peptides that specifically modulate Lys48-linked ubiquitin chains. *Nat. Chem.* **11**, 644–652 (2019).
