# Supplementary material for: Selective macrocyclic peptide modulators of Lys63-linked ubiquitin chains disrupt DNA damage repair
Source: Nat Commun. 2022 Oct 18;13:6174. doi: 10.1038/s41467-022-33808-6 (PMC9579194; doi:10.1038/s41467-022-33808-6)
Supplement: Supplementary file 2 — Reporting Summary [file 41467_2022_33808_MOESM2_ESM.pdf]

nature portfolio

Corresponding author(s): Hiroaki Suga and Ashraf Brik

Last updated by author(s): Sep 21, 2022

Reporting Summary

Nature Portfolio wishes to improve the reproducibility of the work that we publish. This form provides structure for consistency and transparency in reporting. For further information on Nature Portfolio policies, see our Editorial Policies and the Editorial Policy Checklist.

Statistics

For all statistical analyses, confirm that the following items are present in the figure legend, table legend, main text, or Methods section.

n/a	Confirmed	
☐	☒	The exact sample size (*n*) for each experimental group/condition, given as a discrete number and unit of measurement
☐	☒	A statement on whether measurements were taken from distinct samples or whether the same sample was measured repeatedly
☐	☒	The statistical test(s) used AND whether they are one- or two-sided<br>*Only common tests should be described solely by name; describe more complex techniques in the Methods section.*
☐	☒	A description of all covariates tested
☐	☒	A description of any assumptions or corrections, such as tests of normality and adjustment for multiple comparisons
☐	☒	A full description of the statistical parameters including central tendency (e.g. means) or other basic estimates (e.g. regression coefficient) AND variation (e.g. standard deviation) or associated estimates of uncertainty (e.g. confidence intervals)
☐	☒	For null hypothesis testing, the test statistic (e.g. *F*, *t*, *r*) with confidence intervals, effect sizes, degrees of freedom and *P* value noted<br>*Give P values as exact values whenever suitable.*
☒	☐	For Bayesian analysis, information on the choice of priors and Markov chain Monte Carlo settings
☒	☐	For hierarchical and complex designs, identification of the appropriate level for tests and full reporting of outcomes
☒	☐	Estimates of effect sizes (e.g. Cohen's *d*, Pearson's *r*), indicating how they were calculated

Our web collection on statistics for biologists contains articles on many of the points above.

Software and code

Policy information about availability of computer code

Data collection	LCQ Fleet mass spectrometer (Thermo Scientific) with an ESI source was used to perform electrospray ionization mass spectrometry (ESI-MS). An infinite M200 fluorescence plate reader (TECAN) was used for fluorescent measurements. Live cell CLSM images were captured using Confocal Zeiss LSM 710 equipped with 40× NA 1.2 water immersion objective lens using a 1 AU pinhole settings. Blots were imaged in the Fusion-400 ECL detection system. cells with comets were taken by a fluorescence microscope (Axio Observer Z1 LSM 700, Zeiss) with a 63x Plan-APOCHROMAT 63X/1.4 oil DTC objective (Zeiss) and a camera (AxioCam MRm, Zeiss). The different populations of cells were analyzed using a CYTEK Aurora flow cytometer. Mass spectrometry for proteomics study was performed by Q Exactive plus mass spectrometer (Thermo Fisher)
Data analysis	CLSM images were analyzed using ZEN 3.2 (blue edition). The tail moment intensity profile in the comet assay was analyzed using the “OpenComet” software plugged-in to FiJi (ImageJ2,open access). The unmixed data files from CYTEK Aurora were analyzed using FCS Express software (version 6). The mass spectrometry data of proteomics study was analyzed using the MaxQuant software (version 1.5.2.8).<br>IB quantification was performed using FiJi (ImageJ2,open access) and Graphpad software (Prism 8 version), For proteomics volcano plot analysis Perseus software (versions 1.6.7.0), Figures in the main text prepared in Adobe Illustrator (Version 2020)

For manuscripts utilizing custom algorithms or software that are central to the research but not yet described in published literature, software must be made available to editors and reviewers. We strongly encourage code deposition in a community repository (e.g. GitHub). See the Nature Portfolio guidelines for submitting code & software for further information.

Data

Policy information about availability of data

All manuscripts must include a data availability statement. This statement should provide the following information, where applicable:

- Accession codes, unique identifiers, or web links for publicly available datasets
- A description of any restrictions on data availability
- For clinical datasets or third party data, please ensure that the statement adheres to our policy

The mass spectrometry proteomics data have been deposited to the ProteomeXchange Consortium via the PRIDE partner repository with the dataset identifier PXD035924 [https://www.ebi.ac.uk/pride/archive/projects/PXD035924]. All other related data are available from the corresponding authors upon reasonable request. All source data are provided as a Source data file. Uncropped and unprocessed scans of the most important blots are provided in the Source data.

Human research participants

Policy information about studies involving human research participants and Sex and Gender in Research.

Reporting on sex and gender	N/A
Population characteristics	N/A
Recruitment	N/A
Ethics oversight	N/A

Note that full information on the approval of the study protocol must also be provided in the manuscript.

Field-specific reporting

Please select the one below that is the best fit for your research. If you are not sure, read the appropriate sections before making your selection.

[x] Life sciences [ ] Behavioural & social sciences [ ] Ecological, evolutionary & environmental sciences

For a reference copy of the document with all sections, see nature.com/documents/nr-reporting-summary-flat.pdf

Life sciences study design

All studies must disclose on these points even when the disclosure is negative.

Sample size	No sample size calculation was performed prior to experiments. However, in each case sample size was appropriate based on the consistency of measurable differences between groups. To estimate the statistically significant data we chosen n of at least 3 in most of the experiments.
Data exclusions	No data were excluded from analysis.
Replication	All experiments performed in this study were reliably reproducible. Information about the number of replicates is indicated in the relevant figure legends.
Randomization	We have not performed any randomization techniques for any of our experiments since it was not applicable due to the nature of our study. Samples were distributed based on the treatments/experimental conditions applied and their identity was known befor experimental set up and data analysis.
Blinding	The investigators were blinded for proteomics Mass-spectrometry data collection and in a few cases for data analysis wherever necessary. For other experiments the groups/sets/samples can not be blinded since they are easily identifiable due to their distinct properties.

Reporting for specific materials, systems and methods

We require information from authors about some types of materials, experimental systems and methods used in many studies. Here, indicate whether each material, system or method listed is relevant to your study. If you are not sure if a list item applies to your research, read the appropriate section before selecting a response.

Materials & experimental systems

n/a	Involved in the study
☐	☒ Antibodies
☐	☒ Eukaryotic cell lines
☒	☐ Palaeontology and archaeology
☒	☐ Animals and other organisms
☒	☐ Clinical data
☒	☐ Dual use research of concern

Methods

n/a	Involved in the study
☒	☐ ChIP-seq
☐	☒ Flow cytometry
☒	☐ MRI-based neuroimaging

Antibodies

Antibodies used	Rabbit monoclonal phospho-Histone H2A.X (phospho Ser139): Phospho-Histone H2A.X (Ser139) (20E3) Rabbit mAb, 1:1500, Cell Signaling Technology, 9718, 20E3<br>Rabbit monoclonal Histone H2A.X: Recombinant Anti-Histone H2A.X antibody [EPR895] (ab124781), 1:1500, Abcam, ab124781, EPR895<br>Rabbit monoclonal Ubiquitin (linkage-specific Lys63): Anti- Ubiquitin (linkage-specific K63) antibody (EPR8590-448), 1:2000, Abcam ab179434, EPR8590-448<br>Rabbit monoclonal Ubiquitin (linkage-specific Lys48): Anti- Ubiquitin (linkage-specific K48) antibody [EP8589], 1:1500, Abcam, ab140601, EP8589<br>Mouse monoclonal Ubiquitin (P4D1): Ubiquitin (P4D1) sc-8017 1:1000, Santa Cruz Biotechnology, Sc-8017<br>Mouse monoclonal FLAG M2: Monoclonal ANTI-FLAG® M2 antibody produced in mouse, 1:1000, Sigma, F1804 M2<br>Secondary goat anti-rabbit IgG H&L(HRP):Goat anti-rabbit IgG H&L (HRP), 1:10000, Abcam, ab6721<br>Secondary goat anti-mouse IgG H&L(HRP): Goat anti-rabbit IgG H&L (HRP) 1:10000, Abcam, ab6728
Validation	The validation reports of all commercially available antibodies are provided in a database of manufacturer's website.

Eukaryotic cell lines

Policy information about cell lines and Sex and Gender in Research

Cell line source(s)	HeLa-CCL-2™ HEK293T-CRL-3216™, and U-2 OS-HTB-96™ were purchased from ATCC.
Authentication	Cell lines were not authenticated by ourselves.
Mycoplasma contamination	All cell lines were tested to be Mycoplasma negative.
Commonly misidentified lines (See ICLAC register)	None of the cell lines used in the study were found in Commonly misidentified line database

Flow Cytometry

Plots

Confirm that:

☒ The axis labels state the marker and fluorochrome used (e.g. CD4-FITC).

☒ The axis scales are clearly visible. Include numbers along axes only for bottom left plot of group (a 'group' is an analysis of identical markers).

☒ All plots are contour plots with outliers or pseudocolor plots.

☒ A numerical value for number of cells or percentage (with statistics) is provided.

Methodology

Sample preparation	All the samples were prepared as manufacturer's protocol (provided in Supporting Information), In brief, sample preparation for Apoptosis study): Apoptotic cell death was estimated by using the standard MEBCYTO® Apoptosis Kit (MBL) protocol. In brief, HeLa cells, seeded on were treated with samples (1 µM of peptide 2 or DMSO) for 96 h at 37 °C with 5% CO2. After sample treatment, the cells were harvested from the 60 mm dish by trypsinization and centrifuge at 2.01X10-4 g for 4 mins. The cells were washed once with phosphate-buffered saline (PBS, without Mg2+ and Ca2+) and were resuspended in supplied binding buffer subsequently stained with Annexin V-FITC and propidium iodide (PI). The annexin V–FITC positive cells were considered as apoptotic cells moreover, the early and late apoptotic cells were distinguished by negative and positive PI signals, respectively. We could not proceed with a higher concentration of the cyclic peptides due to the solubility issues in the buffer medium.<br>Sample preparation (Cell cycle study): Cell cycle analysis was performed using FxCycle™ PI/RNase Staining Solution. HeLa cells were treated similarly as previously (discussed above). Following detachment, the cells were washed twice with cold PBS and then fixed with 70% ice-cold ethanol at −20°C overnight. The fixed cell pellets were washed twice with cold PBS and then incubated with PI/RNase staining solution on ice for 30 min in the dark.

Instrument	CYTEK Aurora
Software	SPECTROFLO®SOFTWARE (version 3.0.3)
Cell population abundance	20,000 cells were taken for each samples
Gating strategy	For acquisition, obtain spectral information by moving the polygon gate on the FSC vs SSC plot to include the population of interest and removes cellular debris during the analysis and FSC-A versus FSC-H plot removes the cell doublets during the analysis. SPECTROFLO®SOFTWARE (version 3.0.3) sets the default gate near or on the peak emission channel. All are consistent throughout the experiment.

☒ Tick this box to confirm that a figure exemplifying the gating strategy is provided in the Supplementary Information.
